# Platinum-based drug-induced depletion of amino acids in the kidneys and liver

**DOI:** 10.3389/fonc.2022.986045

**Published:** 2022-09-21

**Authors:** Katerina Mitrevska, Natalia Cernei, Hana Michalkova, Migue Angel Merlos Rodrigo, Ladislav Sivak, Zbynek Heger, Ondrej Zitka, Pavel Kopel, Vojtech Adam, Vedran Milosavljevic

**Affiliations:** ^1^ Department of Chemistry and Biochemistry, Mendel University in Brno, Brno, Czechia; ^2^ Central European Institute of Technology, Brno University of Technology, Brno, Czechia; ^3^ Department of Inorganic Chemistry, Faculty of Science, Palacky University, Olomouc, Czechia

**Keywords:** amino acids, chicken embryo, platinum nanoparticles, TCA, methionine cycle, toxicity

## Abstract

Cisplatin (cis-diamminedichloroplatinum II; CDDP) is a widely used cytostatic agent; however, it tends to promote kidney and liver disease, which are a major signs of drug-induced toxicity. Platinum compounds are often presented as alternative therapeutics and subsequently easily dispersed in the environment as contaminants. Due to the major roles of the liver and kidneys in removing toxic materials from the human body, we performed a comparative study of the amino acid profiles in chicken liver and kidneys before and after the application of CDDP and platinum nanoparticles (PtNPs-10 and PtNPs-40). The treatment of the liver with the selected drugs affected different amino acids; however, Leu and Arg were decreased after all treatments. The treatment of the kidneys with CDDP mostly affected Val; PtNPs-10 decreased Val, Ile and Thr; and PtNPs-40 affected only Pro. In addition, we tested the same drugs on two healthy cell lines, HaCaT and HEK-293, and ultimately explored the amino acid profiles in relation to the tricarboxylic acid cycle (TCA) and methionine cycle, which revealed that in both cell lines, there was a general increase in amino acid concentrations associated with changes in the concentrations of the metabolites of these cycles.

## Introduction

CDDP is currently a standard treatment for various cancers (1, 2). The toxicities related to the application of CDDP are usually dose dependent. Overdoses of CDDP significantly increase the percentage of morbidity and/or mortality in patients (3) and the clinical features associated with CDDP overdose include various side effects of therapy, such as nausea and vomiting, renal insufficiency, electrolyte abnormalities, ototoxicity, peripheral neuropathy, hepatotoxicity, and retinopathy (4–10). DNA repair has a crucial role in controlling the negative side effects of platinum drug therapy (11, 12). The ability of cells to recover from DNA damage after treatment with platinum drugs is crucial role in limiting the ability of the drug to induce apoptosis (13, 14). Platinum-based drugs also trigger reactive oxygen metabolism (ROM) and inhibit the activity of antioxidant enzymes, leading to lipid peroxidation and consequent nephrotoxicity (15, 16). Moreover, nephrotoxicity induced by platinum drugs has been very well documented in clinical oncology. On the other hand, hepatotoxicity has rarely been characterized and is less studied (17, 18). Generally, the liver toxicity of platinum-based drugs can be characterized by elevation of serum transaminases and, less frequently, by mild elevation of serum alkaline phosphatase, LDH, bilirubin and c-glutamyl transpeptidase levels (19, 20). The mechanisms of liver damage induced by platinum-based drugs are poorly described. Histological analysis of the liver revealed cytoplasmic changes, especially around the cells of the central vein, as well as hepatocellular vacuolization and sinusoidal dilatations. The presence of apoptotic lesions suggested that platinum-based drugs damage the liver parenchyma (21). Furthermore, all essential and nonessential amino acids undergo interconversions from one amino acid to another and can be converted into various intermediates of different metabolic cycles to maintain the metabolic homeostasis of the organism. However, platinum drugs have been shown to interfere with and alter amino acid metabolism, as well as the intermediates of the related metabolic cycles, such as the TCA cycle and pyruvate metabolism (22). Considering the negative side effects of CDDP, it is necessary to develop a new potential platinum-based drug with high efficiency and low-severity side effects. The application of inorganic nanoparticles can increase the selectivity for tumor tissue while decreasing the side effects (23). PtNPs have been found to show lower cytotoxicity than CDDP and exhibit excellent anticancer and therapeutic properties (24, 25). The excellent properties of PtNPs are reflected in the possibility of surface modification with various bioactive groups (26–28). It has been reported that PtNPs possess the capacity to pass the cell membrane without limitation (29) but also to induce DNA damage and increase cellular glutathione and genotoxic stress (30–32). Due to the many advantages PtNPs possess, their application as therapeutic agents in cancer therapy have rapidly increased, and the exposure to them mainly includes the patients undergoing this therapy. However, PtNPs, as well as other platinum-based drugs such as CDDP, are inevitably released in the ecosystems through the excretion in urine, polluting the environment (33). The additional application of PtNPs in biomedicine as a material used in diagnostics, drug delivery, imaging, and photothermal therapy, only raise the concerns of second-hand exposure to humans and other life forms inhabiting these ecosystems. Some research regarding the toxicological effects of the PtNPs have been reported, mainly in organisms occupying aquatic environment (33, 34), as well as their implication in the disruption of the proper function of the cardiovascular system in mice (35). Nevertheless, the effects of PtNPs and their behavior inside other tissues are still unclear (36–38). Amino acids play a crucial role in the function of the liver, especially branched-chain amino acids (BCAAs) (39), which have a major role in the prognosis of hepatocellular carcinoma (40). However, amino acid metabolism in the population suffering from kidney and liver diseases is still poorly understood (41–43). In our preliminary study, three different types of malignant cells (prostate – LNCaP, breast – MDA-MB-231 and neuroblastoma – GI-ME-N) were tested *in vitro* after treatment with PtNPs modified with polyvinylpyrrolidone with different molecular weights i.e. polyvinylpyrrolidone with average molecular weight 10000 (PtNPs-10) and polyvinylpyrrolidone with average molecular weight 40000 (PtNPs-40) (44). This study is a continuation of an earlier work assessing the cytotoxicity of PtNPs (44). In this study, we assessed the cytotoxicity and compatibility of PtNPs in healthy cell lines (human keratinocytes – HaCaT and human epithelial kidney cells HEK-293) and examined their effect on the content of amino acids, and on the TCA and methionine cycle upon treatment. Furthermore, we focused our investigation on the changes in the content of fundamental amino acids in the liver and kidney of chicken embryos before and after exposure to CDDP using ion-exchange liquid chromatography (IELC) to determine the amino acid concentrations. The most important aim of this study was to compare the content and representation of amino acids in chicken embryos (liver and kidney) before and after treatment with synthesized platinum nanoparticles (PtNPs-10 and PtNPs-40), which are under consideration as a potential replacement for toxic cytostatic CDDP.

## Materials and methods

The method and materials used in the characterization of the samples and studies of the change in amino acid profile are described in detail in the Supporting Information.

## Results

### Characterization of PtNPs properties

PtNPs characterization was conducted after the nanoparticles were modified with two polymers, polyvinylpyrrolidone with a molecular weight of 10000 (PVP-10) and polyvinylpyrrolidone with a molecular weight of 40000 (PVP-40), as capping agents for stabilization of the particles and reduction of the toxicity effects. The morphology, size and zeta potential of the tested platinum nanoparticles were determined by transmission electron microscopy (TEM) and dynamic light scattering (DLS) using a NANO-ZS particle size analyzer. Mostly, the synthetized PtNPs have a spherical shape with an individual particle size of approximately 10 ± 4 nm, as shown in **Figures S1A** and **S1B**, demonstrating the successful synthesis of the nanoparticles. It is clearly visible that most of the nanoparticles have uniform size and good dispersion. However, the TEM images also show that a minor percentage of the particles have poor colloidal stability and tend to form aggregates with other particles. This behavior of Pt nanoparticles modified with PVP was already described as a result of the interfacial interaction of one nanoparticle with another during the self-assembly process triggered by a spontaneous exothermic reaction (45, 46). The formation of PtNPs was also confirmed by DLS to observe the size and potential (**Figures S1A, S1B**). The DLS measurement confirms the results obtained from TEM, where the average size of PtNPs-10 was found to be 10 ± 2 nm, while PtNPs-40 had an average size of 12 ± 3 nm. The zeta potential results revealed that PtNPs-10 had a zeta potential of −10.6 mV (**Figure S1A**), while PtNPs-40 had a zeta potential of −3.5 mV (**Figure S1B**). The low surface potential of the PtNPs arises from the nature of PVP used in surface modification. PVP is a neutral and hydrophilic polymer that interacts with the solvent during the synthesis of PtNPs, resulting in the formation of nanoparticles with low surface potential (47). However, it is obvious that the majority of both PtNPs have a uniform spherical shape with good dispersity, despite the low ζ-potential values.

To clarify the chemical composition of PtNPs stabilized with PVP in the solid state, X-ray photoelectron spectroscopy (XPS) measurements were carried out to investigate the binding energy on different chemical surface species during synthesis. The XPS data analysis revealed the presence of a high-resolution Pt 4f region with two pairs of doublets (Pt 4f7/2 and Pt 4f5/2) in both PtNPs-10 and PtNPs-40 (**Figure S2**). In detail, for PtNPs-10 (**Figure S2A**), three different Pt oxidation states were detected and identified in the spectrum, which was characteristic of zero-valence metallic Pt(0) with the highest-intensity double peaks corresponding binding energies of 70.8 and 74.1 eV (48, 49), the next highest doublets corresponding to Pt(II) oxide with binding energies of 72.2 and 75.5 eV (49, 50) and the lowest-intensity double peaks to Pt(IV) oxide with binding energies of 73.8 and 77.1 eV (50, 51). The calculation of area doublet peaks revealed that 62.2% belonged to zero-valence metallic Pt(0), 24.8% to Pt(II) oxide and 12.9% to Pt(IV) oxide. It is obvious that most nanoparticles were in a metallic state. The remaining nanoparticles were in a different oxidation state, probably due to incomplete reduction of the Pt species during synthesis (52, 53). Similar chemical signatures and oxidation states were obtained for PtNPs-40 (**Figure S2B**). The lowest intense doublet peaks were attributed to zero-valence metallic Pt(0) with binding energies of 70.7 and 74.0 eV and to Pt(IV) oxide with binding energies of 75.8 and 79.1 eV. The highest doublet peaks were attributed to the Pt(II) oxide with binding energies of 73.7 and 77.0 eV. However, the relative percentage of Pt(II) oxide calculated from the area peaks was found to be 55%, while the zero-valence metallic Pt(0) state was 29.4% and Pt(IV) oxide 15.6%.

The high-resolution XPS data showed the presence of the C and N 1s regions in the scanned PtNP samples (**Figure S2**). Comparing the XPS data of PtNPs-10 and PtNPs-40 revealed similarities in the relative intensities and values of peaks, indicating a similar surface modification. The N 1s regions of the XPS spectra of PtNPs-10 and PtNPs-40 contained a characteristic large peak at a binding energy of 399.8 eV (**Figure S2A, S2B**). This peak represented the binding energy of PVP bulk material assigned to the majority of unperturbed nitrogen atoms in the pyrrolidone system (−C−N−) (54). These results clearly suggested the absence of surface bonding between Pt and N atoms. In the case of PtNPs-10, the minor peak at 397.6 eV attributed to metal−N binding was observed. It is reported that the presence of the minor peak at 397.6 eV is associated with impurities (55, 56). The fitting of the 1s XPS spectrum verified the presence of five components in the PtNPs-10 and PtNPs-40 samples. The strongest peak with a binding energy of 285.0 eV was associated with the formation of adventitious carbon created by the polymer chain (−C−C−). The next highest peak at 285.7 eV belonged to the hydrocarbon functionality associated with the polymeric N heterocyclic compound (–CH_2_–). The peak observed at 286.4 eV was assigned to the presence of carbon in the polymer chain with –C–N– connection (57). The binding energy of the peak detected at 288 eV (−C=O−) was associated with the electron emission from the carbon atom of the amide group carbonyl substituent. These results can be interpreted to indicate the direct bonding of carbonyl to the Pt surface, together with charge transfer from the carbonyl group to platinum (56). The remaining peaks with binding energies of 288.8 eV and 290.1 eV indicated the presence of carbonates. The presence of these peaks was probably due to the direct contact between platinum ions and oxygen atoms from the carbonate ions. The results obtained from the C 1s binding energy clearly indicated that PVP was directly bonded to Pt.

### Cytotoxicity and biocompatibility of PtNPs

#### Cell viability (MTT assay)

**Figure 1:**
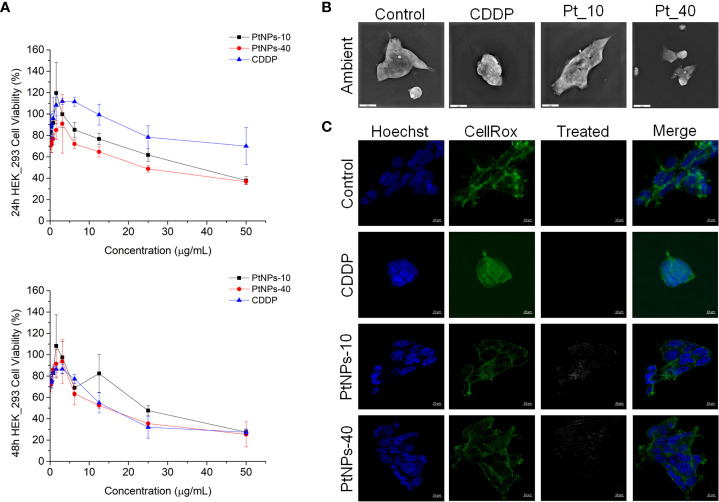
PtNPs surface modification-related toxicity expressed as **(A)** concentration-dependent cell viability of the HEK-293 cell line against PtNPs and CDDP obtained by MTT assay at 24 h and 48 h; **(B)** morphology of HEK-293 cells under a holography and rotational scanning microscope; scale bar 20 μm; and **(C)** laser scanning confocal microscope images of the internalization of PtNPs (white) into the cytoplasm, showing the unaffected morphology of HEK-293 cells. Nuclei (blue: Hoechst nuclei staining) and actin rings (green: phalloidin staining of F-actin); scale bar 10 μm.

**Figure 2:**
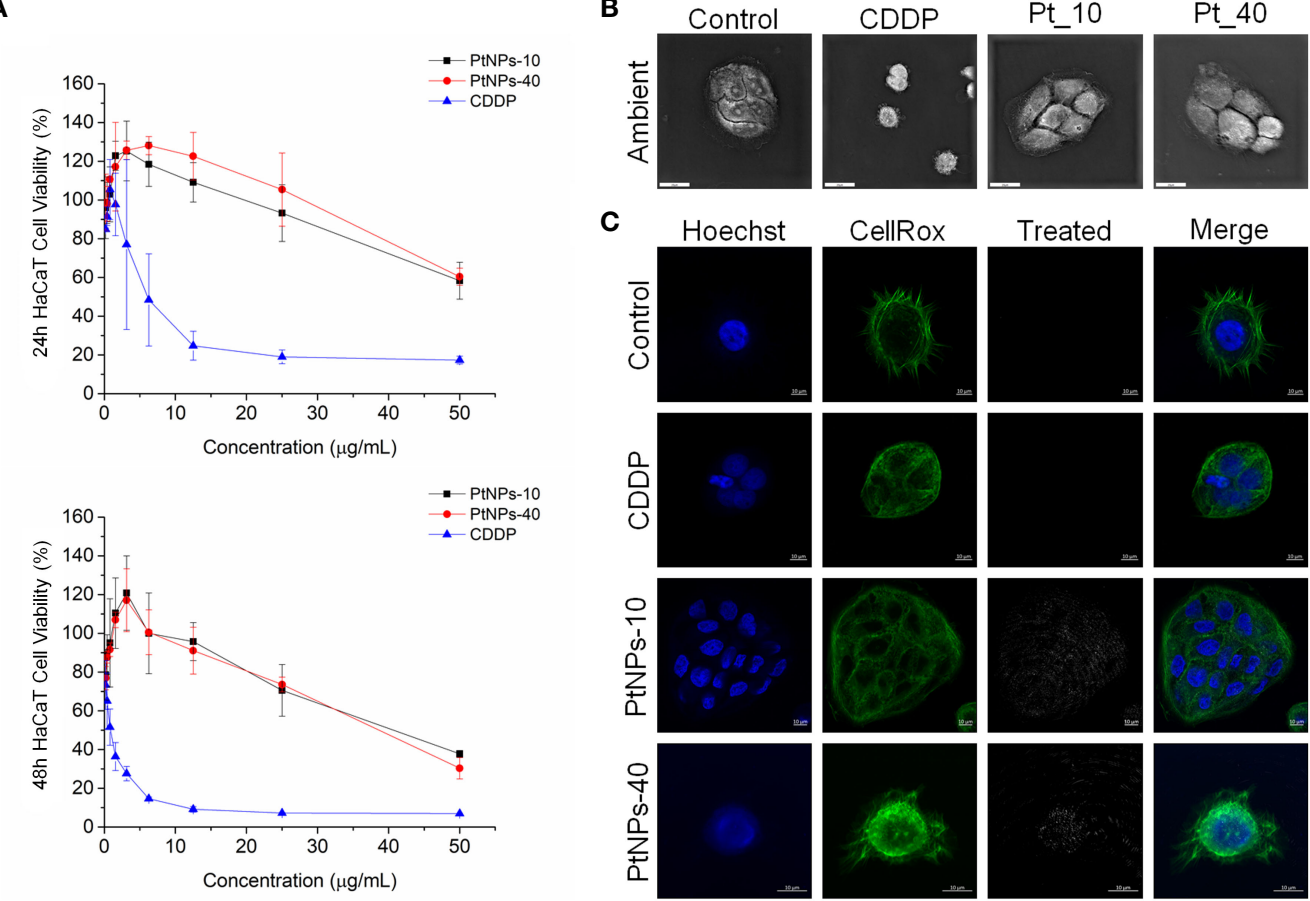
PtNPs surface modification-related toxicity expressed as **(A)** concentration-dependent cell viability of the HaCaT cell line against PtNPs and CDDP obtained by MTT assay at 24 h and 48 h; **(B)** morphology of HaCaT cells under a holography and rotational scanning microscope; scale bar 20 μm; and **(C)** laser scanning confocal microscope images of the internalization of PtNPs (white) into the cytoplasm, showing the unaffected morphology of HaCaT cells. Nuclei (blue: Hoechst nuclei staining) and actin rings (green: phalloidin staining of F-actin); scale bar 10 μm.

In our experiment, *in vitro* testing of the cytotoxic effects of PtNPs-10 and PtNPs-40 was conducted in two different normal human cell lines, HEK-293 and HaCaT. The cytotoxic effects of PtNPs-10 and PtNPs-40 were investigated in a concentration range (0 – 50 µg/mL) and in a time-dependent manner (**Figures 1A**, **2A**) and it was compared with the conventionally used platinum-based drug cytostatic CDDP. The cytotoxicity trend revealed a decrease in cell viability with increasing treatment concentration and with increasing exposure time from 24 h to 48 h. The viability of the HEK-293 cell line indicated a higher sensitivity of the cells than the HaCaT cell line after treatment with PtNPs. PtNPs-10 and PtNPs-40 exhibited cytotoxic effects on the HaCaT cell line during the first 24 h of exposure only at the highest applied concentration (50 μg/mL), reducing the viability of the cells by 42% and 40%, respectively, while the strong cytotoxic effects of CDDP started to appear at a very low concentration (6.25 μg/mL). However, PtNPs and CDDP clearly had higher cytotoxic effects on the HEK-293 cells than on HaCaT cells, and the viability of HEK-293 cells was reduced at lower concentrations than those necessary to reduce the viability of HaCaT cells, with 38% reduced viability upon treatment with 25.0 μg/mL PtNPs-10 and 35% upon treatment with 12.5 μg/mL PtNPs-40. These data confirm the well-known fact that HEK-293 cells are highly sensitive to platinum-based drugs (58). Nonetheless, after 48 h of treatment, the cell lines showed a negligible decrease in viability in comparison with previously described results (**Figures 1A**, **2A**). After the treatment of HEK-293 and HaCaT cell lines with PtNPs-10 and PtNPs-40, a significant decrease in cell viability was observed only at the highest applied concentration, with a 70% decrease in both cell lines. The toxicity of CDDP to HaCaT cell lines after 48 h showed a similar trend after 24 h of treatment, whereas in HEK-293 cells, the cytotoxicity after 48 h seemed more apparent than in the 24 h treatment.

#### Hemocompatibility of PtNPs (Hemolytic assay and protein corona formation)

To observe the interaction of PtNPs with cell membranes and proteins in human blood we performed a hemolytic assay and protein corona test (**Figure S3**). The hemolytic assay was performed in a concentration range from 0–50 μg/mL, for 1 h, and haemolysis degree was calculated from the supernatant absorbance. The hemolytic assay results revealed that PtNPs-10 and PtNPs-40 did not induce hemolysis in comparison with the positive control (0.1% Triton X-100) (**Figures S3A, S3B**). A low level of hemolysis (7%) was observed only at the highest applied concentration of both PtNPs (50 μg/mL). The obtained results indicated good hemocompatibility of the synthetized PtNPs-10 and PtNPs-40.

Since nanoparticles have the ability to interact easily with plasma proteins, we conducted the corona assay to evaluate the potential of PtNPs to form protein coronas through surface adsorption. The protein corona test was conducted at the highest concentration (50 μg/mL) and the quantification of protein corona formation was achieved by SDS-PAGE separation. The assay in plasma protein-rich media revealed that the highest abundance of plasma protein was found in the control plasma sample. As shown in **Figure S3C**, there is absence of larger plasma proteins (>50 kDa) on the surface of PtNPs-10 and PtNPs-40. Keeping in mind that these proteins are present in the plasma sample, this leads to the conclusion that no protein corona formed on the nanoparticle surface.

#### Cell morphology of HEK-293 and HaCaT cells lines upon PtNPs internalization

To visualize the changes in morphology after the treatment with PtNPs and CDDP, as well as the internalization of PtNPs into HEK-293 and HaCaT cells, we obtained images by holography and rotational scanning microscopy and carried out laser scanning confocal fluorescence microscopy analysis after treatment with PtNPs and CDDP (**Figures 1B**, **1C**, **2B**, **2C**). During the observation of the cell morphology of the cells treated with PtNPs, we found no differences in the shape of both cell lines in comparison with untreated cell lines. The cell structures, including the nuclei, seemed to be unaffected by these treatments even after successful internalization of the nanoparticles into the cells. However, in the HaCaT cells, CDDP treatment appeared to cause apoptotic changes (**Figure 2B**).

#### The impact of PtNPs on DNA fragmentation (Comet assay)

To investigate the behavior of our PtNPs after internalization into selected cell lines and the possible occurrence of apoptosis, we performed a comet assay test (DNA damage) and observed the cell line morphology under a fluorescence microscope. The comet assay test was performed at the PtNP IC_50_ concentration calculated from the MTT assay, and the results were collected 24 h after the treatment (**Figure S4A**). Phosphate-buffered saline (PBS) at pH 7.4 was used as a negative control, while hydrogen peroxide (H_2_O_2_) was used as a positive control. The data revealed an absence of DNA damage in the treated cell lines (HEK-293 and HaCaT), clearly showing the same values as the negative control. Collecting and evaluating the data from randomly selected comets, we found that PtNPs do not cause DNA damage in the treated cell lines. The comet tail moment, which represents the level of DNA damage, showed only a low level of damage in the treated cell lines (**Figures S4B, S4C**).

#### PtNPs induced production of ROS

**Figure 3:**
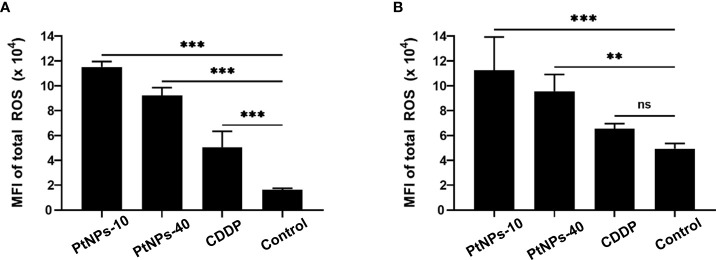
Determination of total ROS induction by PtNPs-10, PtNPs-40 and CDDP treatment. Flow cytometry analysis of ROS induction in HaCaT **(A)** and HEK293 **(B)** cells exposed to 50 µg/mL PtNPs-10, PtNPs-40 or CDDP for 24h. The levels of ROS are shown as MFI ± SD from two independent experiments performed in duplicate. Significant differences to the respective controls are shown (“ns” indicates no significant difference, ^**^p ≤ 0.01, ^***^p ≤ 0.001).

Regarding ROS production, we employed flow cytometry to detect the level of ROS in HEK-293 and HaCaT cell lines after the different treatments. Contrary to Gatto’s findings, our cell lines showed a significantly increased level of ROS production in both cell lines after treatment with PtNPs (**Figure 3**). PtNPs-10 induced the highest ROS production in HEK-293 and HaCaT cells, followed by PtNPs-40. CDDP, on the other hand, showed the lowest increase in ROS in HaCaT cells, but the change was still significant. However, no significant changes in ROS were observed in HEK-293 cells after CDDP treatment.

### *In vitro* study of the effect of platinum-based drugs on HaCaT and HEK-293 cell lines

#### The effect of platinum-based drugs on the amino acid profiles of HaCaT and HEK-293 cell lines

After we investigated the cytotoxic effect of PtNPs, we then analyzed the amino acid content of HaCaT and HEK-293 cell cultures after 24h of threament. Initial analyses were conducted to compare the amino acid content between the two cell lines in the control groups and to assess the difference between the responses of HaCaT and HEK-293 cells to each treatment (**Table S1**). However, since the amino acid contents of untreated HaCaT cells differed significantly from the amino acid contents of untreated HEK-293 cells, further comparison between the responses of the two cell lines to the same treatment was pointless; thus, each cell line was analyzed separately.

The results from one-way ANOVA revealed that all three treatments caused a general increasing trend in the amino acid concentrations in both cell lines, with the exception of a few amino acids, which showed a decreased concentration after the treatment (**Table 1**). For instance, in HaCaT cells, Asp, Ser, Glu, Gly and Ile displayed a significant increase after treatment with CDDP. In contrast to the CDDP treatment, treatment with PtNPs-10 increased Pro but decreased the concentration of Ser in HaCaT cells. The treatment with PtNPs-40 showed a higher resemblance to the CDDP treatment, with an overlap of the affected amino acids. In addition to the increased concentrations of Asp, Ser, Glu, Gly, and Ile, the concentrations of Pro, Ala, Val, Met, and Tyr were also significantly higher than those in the control group, indicating that the treatment affected the majority of amino acids. Comparison of the effects of different drug treatments also revealed significant differences between the amino acids after different treatments. However, the most apparent differences were observed between PtNPs-10 and PtNPs-40 treatment, corresponding to the fact that PtNPs-10 caused significant changes in only two amino acids, whereas PtNPs-40 affected nearly the whole amino acid profile of HaCaT cells.

**Table 1:** Concentration of amino acids in the HaCaT cell line, shown as the mean difference between the treated cells and the control in a 95% confidence interval for the mean difference, ± standard error (S.E.) for each amino acid.

HaCaT	CDDP vs. Control		PtNP-10 vs. Control		PtNP-40 vs. Control		
Amino acid	Mean difference (µmol/L)(95% CI)	p value	Mean difference (µmol/L)(95% CI)	p value	Mean difference (µmol/L) (95% CI)	p value	S.E.
Asp	51.50 (24.48 to 78.52)*	0.001*	26.80 (-0.22 to 53.82)	0.052	116.69 (89.67 to 143.71)*	0.000*	± 8.44
Thr	21.36 (-6.19 to 48.91)	0.138	-1.55 (-29.10 to 26.01)	0.998	24.66 (-2.89 to 52.21)	0.080	± 8.60
Ser	54.50 (16.45 to 92.54)*	0.008*	-49.67 (-87.72 to -11.63)*	0.013*	39.71 (1.67 to 77.75)*	0.041*	± 11.88
Glu	50.96 (22.24 to 79.68)*	0.002*	27.90 (-0.82 to 56.61)	0.057	138.52 (109.80 to 167.23)*	0.000*	± 8.97
Pro	28.25 (-25.67 to 82.17)	0.393	56.44 (2.51 to 110.36)*	0.041*	66.02 (12.09 to 119.94)*	0.019*	± 16.84
Gly	31.38 (0.87 to 61.89)*	0.044*	25.09 (-5.42 to 55.60)	0.112	87.85 (57.34 to 118.35)*	0.000*	± 9.53
Ala	15.65 (-25.83 to 57.12)	0.639	7.74 (-33.74 to 49.21)	0.930	82.86 (41.39 to 124.34)*	0.001*	± 12.95
Cys	20.58 (-33.00 to 74.16)	0.627	3.84 (-49.73 to 57.42)	0.995	-0.94 (-54.52 to 52.64)	1.000	± 16.73
Val	20.36 (-10.20 to 50.91)	0.222	-10.75 (-41.30 to 19.80)	0.685	34.73 (4.17 to 65.28)*	0.027*	± 9.54
Met	15.07 (-7.30 to 37.45)	0.215	6.60 (-15.78 to 28.98)	0.783	34.93 (12.56 to 57.31)*	0.005*	± 6.99
Ile	44.44 (9.61 to 79.26)*	0.015*	19.31 (-15.51 to 54.14)	0.350	94.94 (60.12 to 129.77)*	0.000*	± 10.87
Leu	-11.52 (-57.76 to 34.72)	0.854	-21.26 (-67.50 to 24.98)	0.494	4.08 (-42.16 to 50.31)	0.992	± 14.44
Tyr	18.70 (-7.38 to 44.78)	0.178	5.25 (-20.83 to 31.34)	0.914	28.61 (2.52 to 54.69)*	0.032*	± 8.15
Phe	1.86 (-37.14 to 40.85)	0.999	-6.91 (-45.91 to 32.09)	0.939	15.42 (-23.58 to 54.42)	0.607	± 12.18
His	29.47 (-87.18 to 146.12)	0.849	17.22 (-99.43 to 133.86)	0.963	33.82 (-82.83 to 150.46)	0.791	± 36.43
Lys	0.71 (-20.31 to 21.73)	1.000	-3.60 (-24.62 to 17.42)	0.944	-1.39 (-22.41 to 19.63)	0.996	± 6.56
Arg	-559.21 (-1692.42 to 574.00)	0.440	-439.50 (-1572.70 to 693.71)	0.620	-483.70 (-1616.90 to 649.51)	0.551	± 353.87

A negative sign in front of the mean difference indicates a decrease in the concentration of the amino acid in the cells treated with the selected treatment compared to the untreated cells. Asterisks and bold formatting indicate a statistically significant difference between the treated samples and untreated cells at p ≤ 0.05 (one-way ANOVA, Tukey HSD *post hoc*).

The amino acid profile in HEK-293 cells was slightly different, showing a significant increase in most amino acids after CDDP treatment, except for Pro, Tyr, Lys, and Arg (**Table 2**). In contrast to CDDP, treatment with PtNPs-10 led to significantly higher concentrations of only two amino acids, Cys and Arg, whereas Ser showed similar behavior to that in HaCaT cells, displaying a decreased concentration, which did not occur in the case of the other treatments. However, the application of PtNPs-40 as a treatment resulted in substantially higher concentrations of half of the examined amino acids, including Asp, Thr, Ser, Glu, Ala, Cys, Val, Leu, and His. The rest of the amino acids in both the HaCaT and HEK-293 cell lines showed no significant changes, or they had different reactions to the presence of CDDP, PtNPs-10 or PtNPs-40, being decreased in the presence of one and increased in the presence of the other type of drug. Additionally, the differences between the effects of the different dugs were estimated, showing that the amino acid profile of HEK-293 cells significantly differed after treatment with PtNPs-10 or PtNPs-40 compared to CDDP, whereas PtNPs-10 and PtNPs-40 showed more similar responses for most of the amino acids.

**Table 2:** Concentration of amino acids in the HEK-293 cell line, shown as the mean difference between the treated cells and the control in a 95% confidence interval for the mean difference ± standard error (S.E.) for each amino acid.

HEK-293	CDDP vs. Control		PtNP-10 vs. Control		PtNP-40 vs. Control		
Amino acid	Mean difference (µmol/L)(95% CI)	p value	Mean difference (µmol/L)(95% CI)	p value	Mean difference (µmol/L) (95% CI)	p value	S.E.
Asp	424.01 (208.12 to 639.89)*	0.001*	97.35 (-118.53 to 313.24)	0.509	228.63 (12.74 to 444.51)*	0.038*	± 67.41
Thr	248.62 (192.32 to 304.92)*	0.000*	38.43 (-17.87 to 94.73)	0.207	127.73 (71.43 to 184.03)*	0.000*	± 17.58
Ser	336.73 (222.15 to 451.32)*	0.000*	-149.94 (-264.52 to -35.36)*	0.013*	171.34 (56.75 to 285.92)*	0.006*	± 35.78
Glu	552.50 (372.31 to 732.69)*	0.000*	162.82 (-17.37 to 343.01)	0.077	265.55 (85.36 to 445.74)*	0.007*	± 56.27
Pro	437.51 (-517.01 to 1392.03)	0.497	-76.21 (-1030.74 to 878.31)	0.994	101.89 (-852.63 to 1056.41)	0.985	± 298.07
Gly	343.32 (186.53 to 500.11)*	0.001*	152.92 (-3.87 to 309.71)	0.056	111.52 (-45.27 to 268.30)	0.183	± 48.96
Ala	440.10 (325.45 to 554.75)*	0.000*	88.46 (-26.19 to 203.11)	0.140	217.78 (103.12 to 332.43)*	0.001*	± 35.80
Cys	450.53 (273.78 to 627.27)*	0.000*	219.32 (42.58 to 396.07)*	0.017*	202.04 (25.29 to 378.78)*	0.026*	± 55.19
Val	125.95 (9.02 to 242.89)*	0.035*	33.81 (-83.12 to 150.74)	0.792	116.70 (-0.23 to 233.63)*	0.050*	± 36.51
Met	106.36 (44.09 to 168.62)*	0.003*	50.06 (-12.21 to 112.32)	0.121	57.54 (-4.72 to 119.81)	0.070	± 19.44
Ile	345.08 (183.63 to 506.53)*	0.001*	98.28 (-63.17 to 259.73)	0.282	157.80 (-3.66 to 319.25)	0.055	± 50.42
Leu	146.35 (42.92 to 249.78)*	0.008*	-15.42 (-118.85 to 88.01)	0.962	114.57 (11.14 to 218.00)*	0.031*	± 32.30
Tyr	140.50 (-5.67 to 286.66)	0.060	7.37 (-138.80 to 153.53)	0.998	70.84 (-75.32 to 217.00)	0.454	± 45.64
Phe	162.36 (13.68 to 311.03)*	0.033*	115.34 (-33.34 to 264.01)	0.137	86.48 (-62.20 to 235.15)	0.314	± 46.43
His	305.07 (202.82 to 407.32)*	0.000*	96.57 (-5.68 to 198.82)	0.064	140.48 (38.23 to 242.73)*	0.010*	± 31.93
Lys	56.89 (-18.20 to 131.99)	0.149	32.51 (-42.58 to 107.61)	0.540	50.42 (-24.67 to 125.51)	0.217	± 23.45
Arg	151.09 (-66.97 to 369.14)	0.198	582.07 (364.01 to 800.12)*	0.000*	-68.08 (-286.13 to 149.98)	0.754	± 68.09

A negative sign in front of the mean difference indicates a decrease in the concentration of the amino acid in the cells treated with the selected treatment compared to the untreated cells. Asterisks and bold formatting indicate a statistically significant difference between the treated samples and untreated cells at p ≤ 0.05 (one-way ANOVA, Tukey HSD *post hoc*).

#### Principal component analyses of amino acid profiles in HaCaT and HEK-293 cells

**Figure 4:**
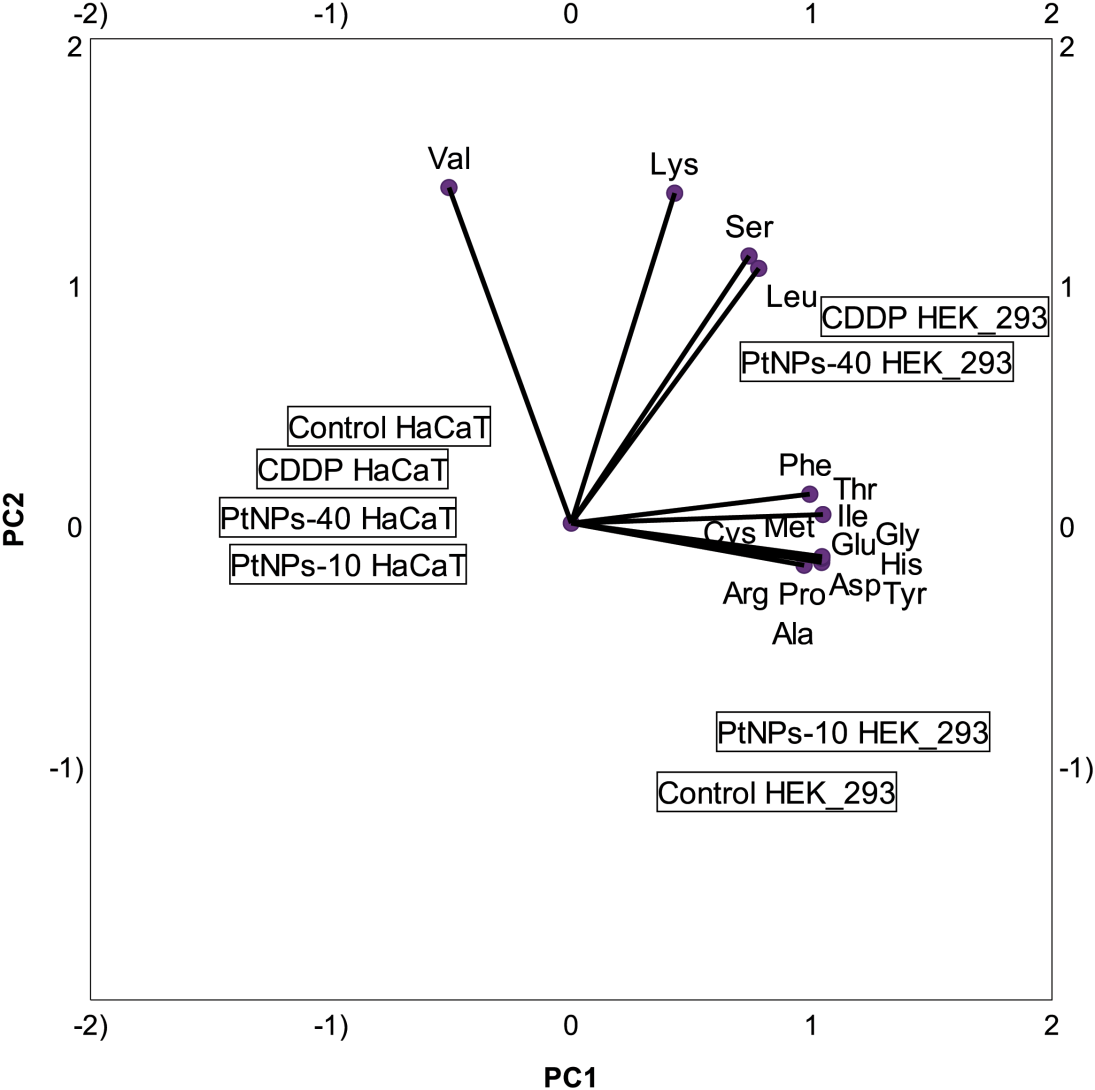
Categorical PCA biplot projection of amino acid behavior in HaCaT and HEK-293 cell lines and the effects of different treatments.

In addition to the linear regression model, categorical principal component analysis was performed to elucidate the relationship between cell lines, treatments and amino acids. A two-factor plane was used, where 83% of the explained variability was attributed to the first component and 14% of explained variability was attributed to the second component. According to the results in **Figure 4**, the HaCaT cells from the four groups (control, CDDP, PtNPs-10 and PtNPs-40) are localized and clustered together on the left side of component 1, clearly distinguishing these cells from the HEK-293 cell line. The treatments of HEK-293 cells, on the other hand, were localized on the right side of the biplot; however, they were separated into two clusters, where PtNPs-10 and the control group belonged to one cluster in the lower right quadrant, and the upper right quadrant was occupied by another cluster containing CDDP and PtNPs-40.

**Figure 5:**
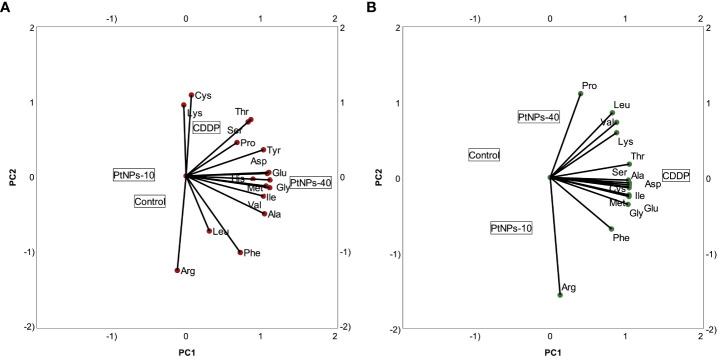
Categorical PCA biplot projection of amino acids and treatments. **(A)** Categorical PCA biplot for HaCaT cell line. **(B)** Categorical PCA biplot for HEK-293 cell line.

To portray the amino acid and treatment relationships in each cell line more clearly, we prepared PCA biplots for each cell line separately. **Figure 5A** shows the biplot for the HaCaT cell line, where PtNPs-40-treated cells are clearly marked as a separate group with the highest concentration of most of the amino acids analyzed. The cells treated with CDDP are another group characterized by the highest concentrations of Lys and Cys and similar concentrations of Ser and Thr compared to PtNPs-40. The control group and cells treated with PtNPs-10 seem to be clustered together, although the proximity of the control group to Arg in the PCA biplot indicates that the control group is distinguished by the highest levels of Arg compared to the other groups. The biplot for HEK-293 cells depicts an even more defined separation of each group (**Figure 5B**). The most obvious differentiation is between CDDP, which was responsible for the highest contents of the majority of amino acids, and the control group, which appears on the opposite side from CDDP. This distinction completely resembles the level of significance obtained from the ANOVAs. The control group, as well as the other two treatments (PtNPs-10 and PtNPs-40), are also defined as separate groups in the biplot, and the group of PtNPs-10-treated cells is characterized by the highest concentration of Arg.

#### Metabolism of amino acids and related cycles in HaCaT and HEK-293 cells

**Figure 6:**
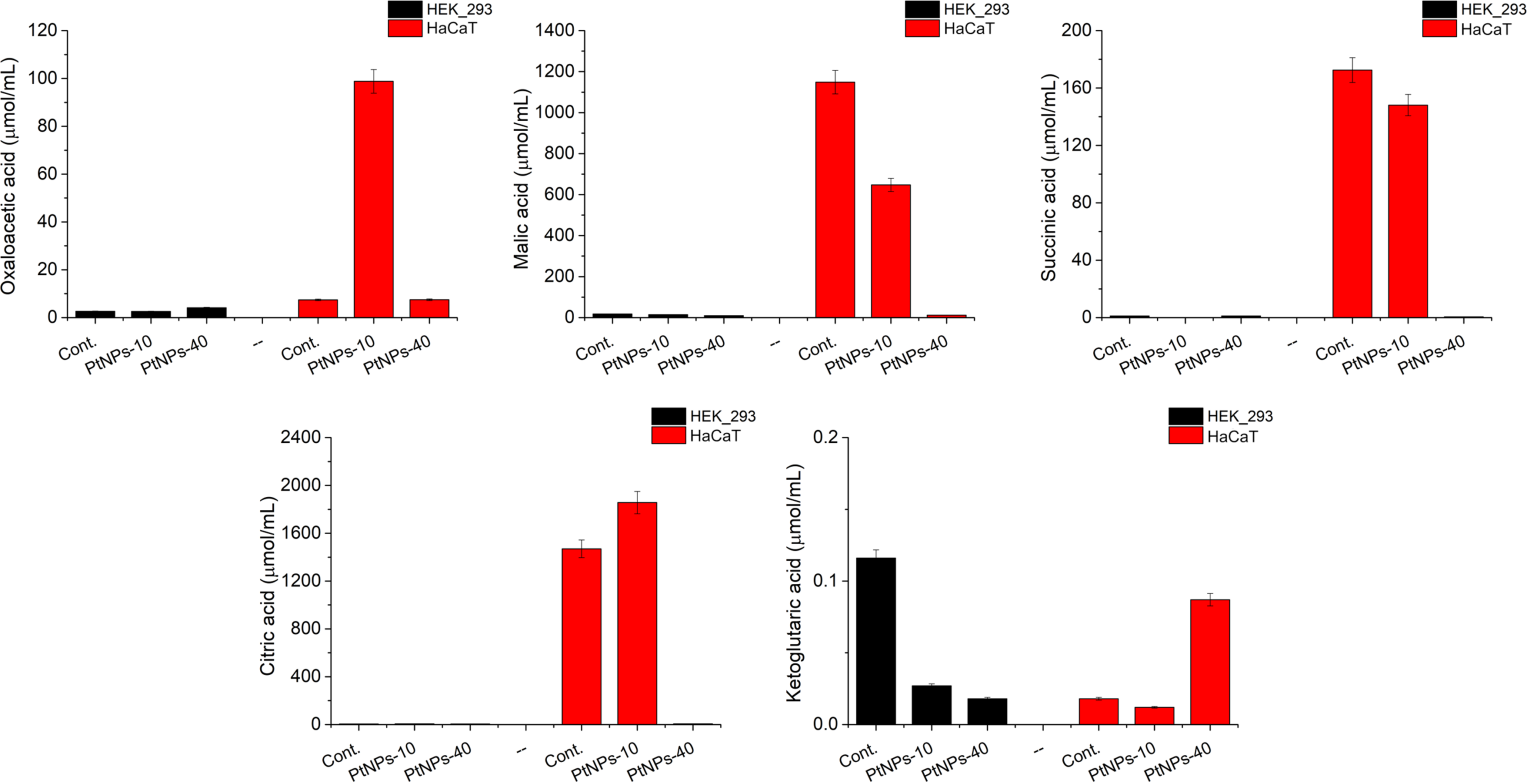
Analysis of selected TCA metabolites produced in HEK-293 and HaCat cell lines treated with PtNPs-10 or PtNPs-40. The charts show the concentration of each metabolite in the untreated control group and after the treatment with PtNPs-10 and PtNPs-40. The concentration of the metabolites is determined by LC–MS/MS.

**Figure 7:**
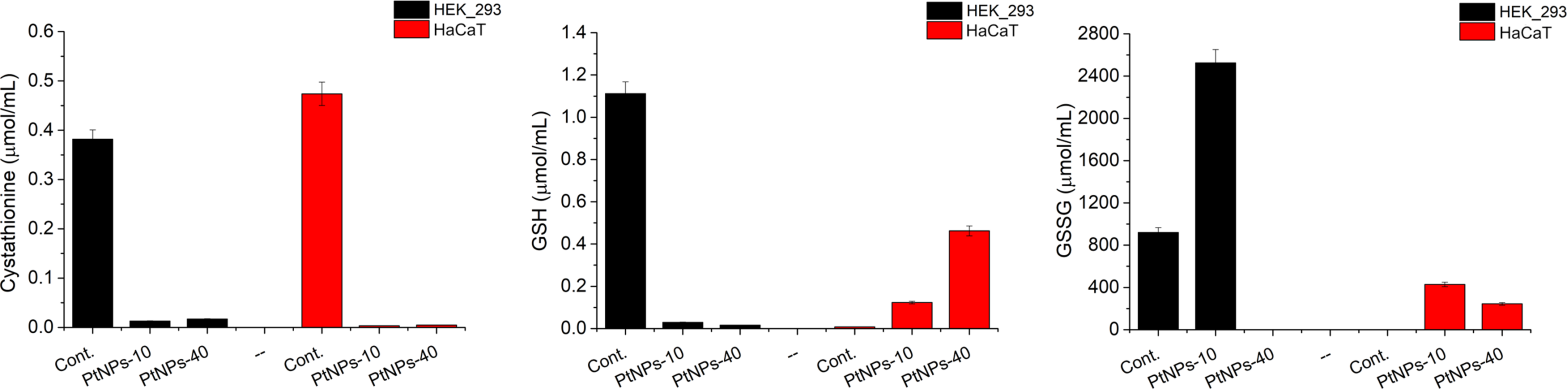
Analysis of selected metabolites related to the methionine cycle produced in HEK-293 and HaCat cell lines treated with PtNPs-10 or PtNPs-40. The charts show the concentration of each metabolite in the untreated control group and after the treatment with PtNPs-10 and PtNPs-40. The concentration of the metabolites is determined by LC–MS/MS.

In relation to the determination of the amino acid contents in treated and untreated HaCaT and HEK-293 cells, we conducted a metabolic investigation of the cells, including the TCA cycle and the methionine cycle (**Figures 6**, **7**). We performed a liquid chromatography analysis of the main TCA cycle intermediates in HaCaT and HEK-293 cells, and we compared their concentration in the untreated cells with the concentrations in the cells treated with PtNPs-10 and PtNPs-40 separately. The analysis was conducted in a brief exploratory manner, to detect weather the amino acids alteration affects the main metabolic cycles as well and vice versa.

Observing the results of the control and PtNPs-10-treated HaCaT cells, we noticed that the production of citric acid was increased at the expense of Ser, whose concentration was significantly decreased (**Figure 6**). Malic acid and oxaloacetic acid showed more noticeable changes, that is, an increase in the concentration of oxaloacetic acid and a decrease in malic acid content. In addition the ketoglutaric acid was slightly decreased.

The treatment of HaCaT cells with PtNPs-40 led to more apparent changes in TCA intermediates than treatment with PtNPs-10 (**Figure 6**). The content of citric acid was reduced substantially despite the abundance of the necessary amino acids. The malic acid concentration was decreased even more substantially than in HaCaT cells treated with PtNPs-10. In contrast to the treatment with PtNPs-10, treatment with PtNPs-40 caused an increase in ketoglutaric acid, showing a relationship between oxaloacetic acid and ketoglutaric acid that is opposite to that found in HaCaT cells treated with PtNPs-10. The treatment with PtNPs-40 also led to significant increases in Val, Met, and Ile, which are responsible for the synthesis of succinyl-CoA; however, succinic acid derived from succinyl-CoA was present in considerably lower amounts than in the control.

The participants of the TCA cycle had considerably lower concentrations in HEK-293 cells than in HaCaT cells; thus, we will can comment only the most apparent deviations of the treated cells compared to the control group (**Figure 6**). The most obvious change in the cells treated with PtNPs-10 was in the contents of ketoglutaric acid, which was decreased. Regarding HEK-293 cells treated with PtNPs-40, less noticeable differences in the TCA intermediates were recorded, despite the more apparent change in their amino acid profile, mostly decreasing ketoglutaric acid, which was even more noticeable than the decrease in the HEK-293 cells treated with PtNPs-10.

The methionine cycle is a multipurpose set of reactions, serving as a source of methyl groups and Cys, a limiting amino acid for the subsequent synthesis of glutathione (GSH) (59). Since GSH is the main respondent against free radicals, we focused our attention on detecting its concentration, as well as observing the behavior of its precursor, cystathionine and the product of GSH depletion, that is its oxidized form GSSG (**Figure 7**). Regarding GSH synthesis, the concentration in HEK-293 cells decreased, whereas in HaCaT cells, GSH levels were elevated upon treatment with both PtNPs. In contrast, the concentration of its precursor, cystathionine, drastically decreased after both treatments in both cell lines. The increase in GSSG concentrations in HEK-293 cells treated with PtNPs-10 proved that the GSH/GSSG neutralization reactions of ROS are enforced as well. However, the treatment with PtNPs-40 led to dubious results with barely detectable GSSG. In addition, both treatments of HaCat cells induced and increase in GSSG levels, along with the increased concentrations of GSH.

### *Ex vivo* studies of the effect of platinum-based drugs in chicken embryos

The *in vitro* effects of different PtNPs treatments on different cell lines showed contrasting results in the cell lines subjected to the treatments, as well as variability among different amino acids under the selected treatments. This led to the presumption that tissue analyses could show similar variances. To investigate the amino acid responses *ex vivo*, we chose the liver and kidneys of chicken embryos as the main sites of amino acid metabolism and distribution. The levels of the selected amino acids in the untreated tissues (control group) and tissues treated with CDDP, PtNPs-10 or PtNPs-40 were determined using IELC.

#### The effect of platinum-based drugs on the weight of chicken embryos and selected organs (liver and kidney)

Prior to the amino acid analyses, we determined the weights of the chicken embryos and the selected organs of the treated groups and compared them to those of the control group (**Table S2**). There was a significant difference between the embryo weights of chickens treated with any form of platinum compared to the control. The weights of embryos treated with the different platinum forms did not differ significantly. Next, the effect on organ weight was analyzed. It was found that the weight of livers was significantly affected by the presence of treatment (control vs. CDDP), as well as by the presence of PtNPs-10 or PtNPs-40 vs. CDDP. The weight of the livers of PtNP-treated embryos was significantly higher but did not reach the weight of the controls. The weight in kidneys differed significantly after all treatments compared to the control; however, no similar trends were observed among different treatments in kidney tissue.

#### The effect of platinum-based drugs on the amino acid profiles of liver and kidneys of chicken embryos

The *ex vivo* effects to the amino acid profile of liver and kidney were examined following the injection of the embryos with the selected treatment (CDDP, PtNPs-10 or PtNPs-40) on the 7^th^ day of development, and the amino acid profiles in the kidneys and liver were analyzed by IELC after 10 days of incubation. In the first step of the analysis, we needed to establish a baseline of amino acid patterns between the liver and kidneys; the untreated tissues were analyzed for this purpose and to assess the difference between the responses of the two organs after each treatment. While there were no significant differences in the amino acid concentrations of the liver and kidneys of the control group, the results of the comparison between liver and kidney treated with a chosen drug revealed that some amino acids in the two organs reacted differently to the same treatment (**Table S1**). Due to the differences in amino acid levels in the selected organs, the effects of the treatments with CDDP, PtNPs-10 and PtNPs-40 were analyzed independently for each organ.

Analyzing the statistical data from the general regression model, we can discuss the changes in the levels of individual amino acids in the tissues, whether a decrease or an increase in their concentration occurred compared to that in the untreated tissue (**Table 3**). For instance, CDDP induced a significant decrease in liver Asp, Ser, Leu, Lys and Arg; however, no significant increase in any amino acids was observed. The PtNPs-10 treatment in the same tissue caused the most prominent effects, leading to depletion of Asp, Glu, Gly, Ala, Val, Leu, Lys and Arg. Interestingly, the treatment had an opposite effect on Cys in the same tissue, resulting in significantly higher levels than in the untreated sample. This finding was observed only in liver tissue, and PtNPs-10 was the only treatment demonstrating a statistically significant elevation in the amino acid levels. Treatment with PtNPs-40 showed the least apparent alterations in the amino acid profile in the liver, decreasing the levels of only three amino acids, Ala, Leu and Arg.

**Table 3:** Concentration of amino acids in liver tissue, shown as the mean difference between the treated cells and the control in a 95% confidence interval for the mean difference, ± standard error (SE) for each amino acid.

Liver	CDDP vs. Control		PtNP-10 vs. Control		PtNP-40 vs. Control	
Amino acid	Mean difference (µmol/L)(95% CI)	SE	Mean difference (µmol/L)(95% CI)	SE	Mean difference (µmol/L)(95% CI)	SE
Asp	-11.06 (-20.92 to -1.20)*	± 4.86	-10.34 (-20.70 to 0.03)*	± 5.10	-8.14 (-18.15 to 1.87)	± 4.93
Thr	-6.33 (-13.14 to 0.47)	± 3.35	-4.07 (-11.21 to 3.08)	± 3.52	-2.81 (-9.72 to 4.09)	± 3.40
Ser	-6.97 (-13.24 to -0.70)*	± 3.09	-4.62 (-11.20 to 1.97)	± 3.24	-4.17 (-10.53 to 2.19)	± 3.13
Glu	-9.04 (-19.75 to 1.68)	± 5.28	-13.76 (-25.02 to -2.50)*	± 5.55	-6.46 (-17.34 to 4.41)	± 5.36
Pro	-3.05 (-8.67 to 2.58)	± 2.77	-4.95 (-10.86 to 0.96)	± 2.91	-5.50 (-11.21 to 0.21)	± 2.81
Gly	-2.45 (-6.43 to 1.53)	± 1.96	-6.17 (-10.35 to -1.98)*	± 2.06	-2.56 (-6.60 to 1.49)	± 1.99
Ala	-4.09 (-9.02 to 0.84)	± 2.43	-7.69 (-12.86 to -2.51)*	± 2.55	-5.02 (-10.02 to -0.01)*	± 2.46
Cys	2.78 (-10.71 to 16.26)	± 6.64	16.00 (1.83 to 30.17)*	± 6.98	8.67 (-5.03 to 22.36)	± 6.74
Val	-5.22 (-11.88 to 1.44)	± 3.28	-11.84 (-18.84 to -4.85)*	± 3.44	-5.76 (-12.51 to 1.00)	± 3.33
Met	-3.67 (-7.38 to 0.04)	± 1.83	-2.00 (-5.90 to 1.89)	± 1.92	-3.06 (-6.83 to 0.70)	± 1.85
Ile	-0.61 (-9.78 to 8.57)	± 4.52	7.25 (-2.38 to 16.89)	± 4.75	2.16 (-7.16 to 11.47)	± 4.59
Leu	-10.69 (-20.45 to -0.93)*	± 4.81	-21.81 (-32.06 to -11.55)*	± 5.05	-11.12 (-21.03 to -1.21)*	± 4.88
Tyr	0.07 (-6.28 to 6.43)	± 3.13	1.35 (-5.33 to 8.02)	± 3.29	0.58 (-5.87 to 7.03)	± 3.18
Phe	-5.83 (-15.48 to 3.83)	± 4.76	1.69 (-8.45 to 11.83)	± 5.00	-2.14 (-11.94 to 7.66)	± 4.83
His	-0.86 (-8.06 to 6.35)	± 3.55	5.19 (-2.38 to 12.76)	± 3.73	2.19 (-5.12 to 9.51)	± 3.60
Lys	-9.63 (-17.53 to -1.73)*	± 3.89	-10.56 (-18.85 to -2.26)*	± 4.09	-5.31 (-13.33 to 2.70)	± 3.95
Arg	-11.38 (-17.88 to -4.88)*	± 3.20	-14.43 (-21.26 to -7.60)*	± 3.36	-9.16 (-15.76 to -2.56)*	± 3.25

A negative sign in front of the mean difference indicates a decrease in the concentration of the amino acid after the selected treatment compared to the control. Asterisks and bold formatting indicate a statistically significant difference between the treated samples and untreated cells at p ≤ 0.05 (one-way ANOVA, Tukey HSD *post hoc*).

Regarding the treatment of kidney tissue with any platinum drug, the results showed that only a few amino acids were affected (**Table 4**). Namely, CDDP decreased Val, and PtNPs-10 treatment decreased the levels of Val, Ile and Thr, whereas PtNPs-40 affected only one amino acid, decreasing the Pro concentration in the kidneys. Additionally, no treatment increases the amino acids in the kidneys. An interesting finding was the contrasting response of Ile in the kidneys and liver: PtNPs-10 treatment decreased the levels of Ile in the kidney but increased the concentration of Ile in the liver; however, the results were on the border of significance or were not deemed significant. Nevertheless, in summary, these results indicate that the amino acid concentrations in the kidneys are less responsive to treatment with platinum drugs than those in the liver.

**Table 4:** Concentration of amino acids in kidney tissue, shown as the mean difference between the treated cells and the control in a 95% confidence interval for the mean difference, ± standard error (SE) for each amino acid.

Kidney	CDDP vs. Control		PtNP-10 vs. Control		PtNP-40 vs. Control	
Amino acid	Mean difference (µmol/L)(95% CI)	SE	Mean difference (µmol/L)(95% CI)	SE	Mean difference (µmol/L)(95% CI)	SE
Asp	-2.53 (-10.31 to 5.24)	± 3.83	-6.89 (-14.92 to 1.15)	± 3.96	-5.25 (-13.14 to 2.65)	± 3.89
Thr	-4.17 (-8.77 to 0.44)	± 2.27	-6.50 (-11.25 to -1.74)*	± 2.34	-4.00 (-8.67 to 0.67)	± 2.30
Ser	-1.79 (-6.91 to 3.32)	± 2.52	-4.68 (-9.96 to 0.61)	± 2.60	-4.34 (-9.53 to 0.86)	± 2.56
Glu	-1.75 (-10.54 to 7.04)	± 4.33	-6.86 (-15.94 to 2.23)	± 4.47	-4.52 (-13.44 to 4.41)	± 4.40
Pro	-2.23 (-8.08 to 3.61)	± 2.88	-5.65 (-11.68 to 0.39)	± 2.97	-6.02 (-11.95 to -0.09)*	± 2.92
Gly	-0.89 (-4.50 to 2.72)	± 1.78	-2.84 (-6.57 to 0.89)	± 1.84	-3.04 (-6.71 to 0.63)	± 1.81
Ala	-1.01 (-5.01 to 2.99)	± 1.97	-3.74 (-7.87 to 0.40)	± 2.04	-2.13 (-6.19 to 1.93)	± 2.00
Cys	1.54 (-11.89 to 14.97)	± 6.61	-2.29 (-16.17 to 11.58)	± 6.83	6.27 (-7.36 to 19.90)	± 6.71
Val	-4.51 (-8.57 to -0.45)*	± 2.00	-4.59 (-8.78 to -0.39)*	± 2.07	-2.69 (-6.81 to 1.44)	± 2.03
Met	-2.25 (-5.00 to 0.49)	± 1.35	-2.65 (-5.49 to 0.18)	± 1.40	-1.70 (-4.48 to 1.09)	± 1.37
Ile	0.03 (-7.56 to 7.62)	± 3.74	-7.82 (-15.66 to 0.02)*	± 3.86	-4.83 (-12.53 to 2.88)	± 3.79
Leu	-6.40 (-13.90 to 1.10)	± 3.70	-4.63 (-12.38 to 3.13)	± 3.82	-6.13 (-13.74 to 1.49)	± 3.75
Tyr	-0.20 (-5.82 to 5.43)	± 2.77	-0.51 (-6.32 to 5.30)	± 2.86	0.47 (-5.24 to 6.18)	± 2.81
Phe	-3.12 (-11.18 to 4.94)	± 3.97	-7.53 (-15.86 to 0.79)	± 4.10	-4.86 (-13.04 to 3.32)	± 4.03
His	3.85 (-1.53 to 9.24)	± 2.65	-2.21 (-7.77 to 3.36)	± 2.74	-0.78 (-6.24 to 4.69)	± 2.69
Lys	-2.75 (-10.00 to 4.50)	± 3.57	-5.40 (-12.90 to 2.09)	± 3.69	-2.32 (-9.68 to 5.04)	± 3.63
Arg	-4.21 (-9.21 to 0.80)	± 2.47	-4.34 (-9.51 to 0.83)	± 2.55	-3.25 (-8.34 to 1.83)	± 2.50

A negative sign in front of the mean difference indicates a decrease in the concentration of the amino acid after the selected treatment compared to the control. Asterisks and bold formatting indicate a statistically significant difference between the treated samples and untreated cells at p ≤ 0.05 (one-way ANOVA, Tukey HSD *post hoc*).

In addition, we compared the individual types of treatments in each organ separately and found that the greatest difference was between the CDDP and PtNPs-10 treatments, and the difference was more significant in the liver than in the kidneys. On the other hand, the differences between CDDP and PtNPs40 and between PtNPs-10 and PtNPs-40 were nonsignificant, or only a few acids in both the liver and kidneys were affected.

#### Principal component analysis of amino acid contents of chicken liver and kidneys

**Figure 8:**
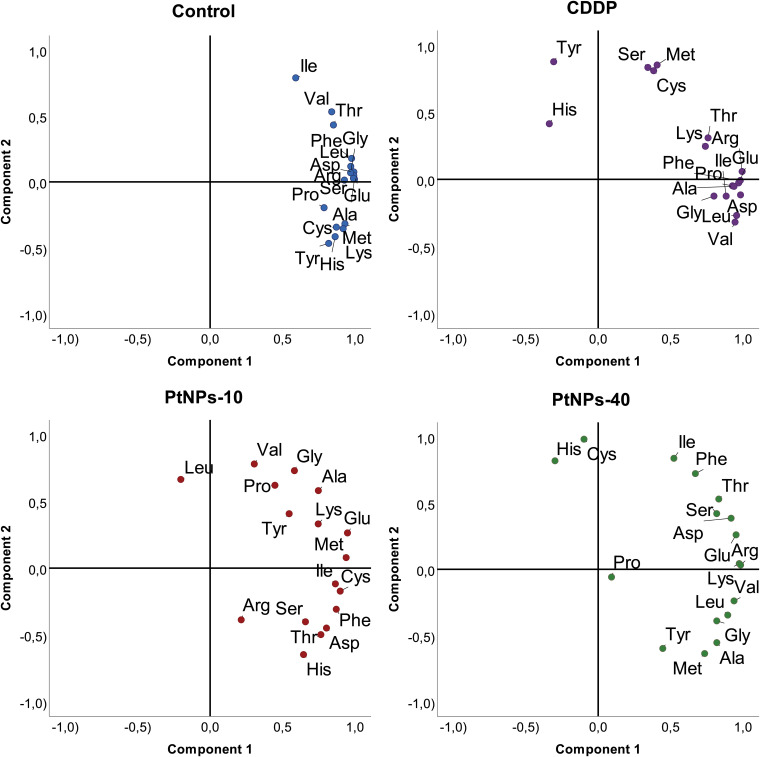
PCA projection of amino acid behavior in liver tissue and the effects of different platinum treatments.

While general linear model results provide powerful insight into each amino acid increase/decrease upon treatment with the selected platinum drug, PCA analyses will provide a more structured perspective, highlighting the most prominent deviations of the analyzed amino acids. Namely, in the PCA graph, CDDP caused the concentration of Ser in the liver to diverge from the main cluster; however, its behavior resembled the behaviors of Cys and Met (**Figure 8**). His and Tyr also followed this trend, although there were obvious differences compared to the first group of Cys, Met and Ser. PtNPs-10 treatment of liver showed a noticeable distinction of Leu, with a close resemblance to the behavior of Val, which was fully consistent with the results obtained from correlation analyses. In addition to Leu and Val, Arg was slightly displaced from the rest of the amino acids; however, all of the amino acids had a more scattered PCA profile than in the control group. Regarding PtNPs-40 treatment, the PCA analyses clearly isolated Pro as an amino acid with completely different behavior from the rest, and a small cluster of His and Cys was separated from the more loosely packed main cluster.

**Figure 9:**
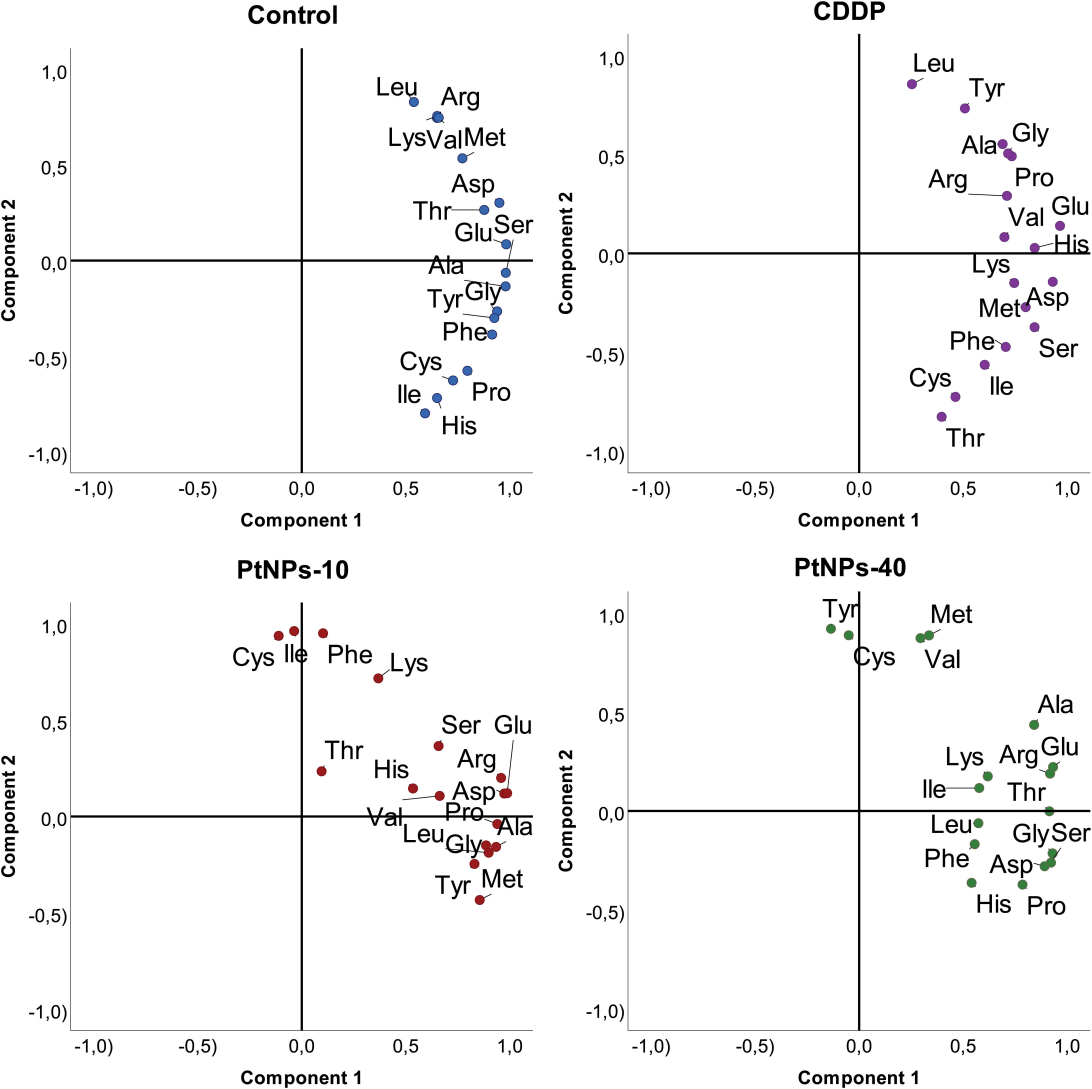
PCA projection of amino acid behavior in kidney tissue and the effects of different platinum treatments.

As indicated by the regression analyses of the data, the kidneys showed a lower response to platinum treatment than liver tissue, and PCA analyses confirmed these initial findings (**Figure 9**). Namely, the CDDP treatment did not show any difference from the control group, and no amino acids could be isolated from the rest of the group. In contrast, treatment with PtNPs-10 organized most of the amino acids in a nicely distinguished cluster, whereas Cys, Ile and Phe separated into a well-defined cluster closely related to Lys. Even though the PtNPs-40 treatment showed the least significant differences from the control, the PCA analyses revealed three distinctive clusters, with most of the amino acids in the main cluster. The second cluster combined Met and Val together, whereas Cys and Tyr defined the third and most distinctive cluster, which diverged to the upper left quadrant of the PCA graph.

## Discussion

### Cytotoxicity and compatibility of PtNPs

Ideal nanoparticles with strong anticancer activity should be not only cost-effective, nontoxic, nonimmunogenic, and < 100 nm in diameter but also highly biodegradable/biocompatible to prevent side effects and accumulation of the nanoparticles in healthy tissues and cells (60). Generally, the polymer-based materials used in the surface modification of different nanoparticles increase their biocompatibility and decrease their toxicity (61). Buchtelova et al.(44) reported that PtNPs modified with PVP-10 had a smaller diameter and stronger toxicity than PtNPs modified with PVP-40 in all tested cell lines (PNT1A, HaCaT, LNCaP, MDA-MB-231 and GI-ME-N). These results indicated that the choice of polymer’s molecular weight for nanoparticle surface modification has a main role in activity.

Comparing the data obtained from the MTT assay, hemolytic assay, comet assay and evaluation of the internalization of PtNPs into different cell lines, they revealed that the nanoparticles become toxic after internalization, while the membranes of the treated cell lines are not disrupted. The good hemocompatibility and the absence of protein coronas allows the PtNPs to express their potential without any interruptions (62). Our results suggested that the PtNP mechanism of action and potential toxicity are not connected to membrane damage but to endocytosis and intracellular processes. Li et al. (63) precisely described the uptake of PtNPs by the cells through endocytosis and storage in lysosomes. They also revealed that the internalization of PtNPs into the cell is limited by downregulation of the small GTPases that regulate endocytosis or by defects in endocytosis. It has also been reported that once PtNPs are inside the cell, they start to degrade in the acidic lysosomal environment and release Pt^+2^ ions, which trigger apoptosis by mitochondrial degradation or DNA damage (64). Even though the PtNPs-10 and PtNPs-40 are not specifically selective to a given cell lineage, the presence of the polymer on their surface endows them with high biocompatibility, allowing successful internalization in the cells without membrane damage, and carryout the toxic effect intracellularly. They have been successfully applied as drug delivery carriers for various drugs, e.g. indomethacin, fentanyl, ibuprofen (65–67). Furthermore, the lack of interaction with plasma proteins, increases their plasma-life, allowing them to circulate longer, without causing damage (68). However, since the application of any metallic particles comes with certain adverse effects, we draw our attention to examining the extent of the undesirable consequences to the healthy cells and tissues.

The viability results of the HEK-293 cell line indicated a higher sensitivity of these cells than the HaCaT cell line after the treatment with PtNPs. These data confirm the well-known fact that HEK-293 cells are highly sensitive to platinum-based drugs (58). However, CDDP clearly had lower cytotoxic effects on the HEK-293 cells than on HaCaT cells. These findings are similarly reflected in the changes of the cell morphology which were observed as apoptosis of HaCaT cells upon the treatment with CDDP, but no other treatments caused any cell disruptions on any cell line. Furthermore, we observed an absence of DNA damage in the treated cell lines (HEK-293 and HaCaT) upon all treatments. On the one hand, a damaging effect of PtNPs on DNA was previously described (32); however, Gatto et al. detected uninterrupted RNA transcription in addition to the scavenging ability of PtNPs toward reactive oxygen species (ROS) (69). Contrary to Gatto’s findings, our cell lines showed a significantly increased level of ROS production in both cell lines after treatment with PtNPs. Despite the increased levels of ROS, the comet assay confirmed that the DNA integrity was maintained; however, such changes in the production of ROS could trigger some antioxidant defense mechanisms, with glutathione (GSH) being the one most reported antioxidants activated upon oxidative stress (70).

### The *in vitro* effect of platinum-based drugs on HaCaT and HEK-293 cell lines

#### PtNPs affect the contents of amino acids in HaCaT and HEK-293 cells

Various studies have shown altered amino acid cell metabolism resulting from treatment with platinum drugs. The PCA analyses in our study showed that neither platinum drug induced profound changes in the amino acid content in HaCaT cells, although PtNPs-40-treated cells responded to the treatment by increasing the concentration of most of the amino acids. In comparison, in HEK-293s the amino acid profile was altered by CDDP, which induced a dominant effect on the whole amino acid set, and PtNPs-40 effects were observed to a smaller extent. Nevertheless, in both cell lines, the treatment with PtNPs-10 seemed to have restricted the amino acid increase, which completely resembles the level of significance obtained from the ANOVAs. The difference in the amino acid profile alterations upon PtNPs-10 and PtNPs-40 mainly depends on the molecular weight of the PVP. In fact, various studies have shown that NPs and drugs stabilized with PVP of smaller molecular weight release the drug faster than PVP with higher molecular size, due to the higher surface that provides better interactions with the medium (71). Accordingly, PtNPs-10 should release more Pt^2+^ than PtNPs-40 during a given time interval, and thus affecting the amino acid profile at a different extent. In addition, the higher induction of ROS production upon PtNPs-10 treatment, supports this variation in amino acid profiles (72).

It is evident in our study that Asp, Glu and Ser are the common amino acids affected in both cell lines upon treatment with CDDP and PtNPs-40. The involvement of these amino acids as responses to CDDP treatment shows a degree of agreement; however, controversial findings have been discussed in several studies. Namely, in normal tubular epithelial cells (NRK-52E), the levels of Glu were increased after CDDP treatment; however, Asp was detected at lower concentrations after this treatment (73). In contrast, the level of Glu was decreased in colon cancer cells treated with oxaliplatin (74). This could be attributed to the fact that a different platinum drug was used as a treatment; however, each cell line operates with a unique metabolic machinery, a difference that is manifested even between cell lines from the same tissue, showing different resistance toward a given drug. Such contrast in amino acid profiles was described in resistant and sensitive neuroblastoma cell lines treated with CDDP, where Asp and Ser noted increased levels in the insensitive neuroblastoma cell line, similar to the observations in our cells, whereas the sensitive cell line did not show any significant changes in these amino acids (75). This is related to the survival ability of the cells, which promotes amino acid synthesis as a mechanism for resistance to the given drug. On the other hand, our treatment with PtNPs-10 led to a decrease in Ser in both cell lines, clearly discriminating this treatment from the other treatments, including CDDP treatment of insensitive neuroblastoma cells. In addition to the fact that PtNPs-10 treatment barely affected the amino acid profiles of the cells, it also indicated different routes of activity. An interesting observation was made regarding Cys levels, which were increased only in HEK-293 cells but not in HaCaT cells after all treatments. Similar findings were described by Kim et al. after CDDP treatment of a different kidney cell line (NRK-52E) (73).

#### PtNPs activate GSH mediated antioxidant defense and alter the TCA cycle performance

The TCA cycle, also known as the Krebs or citric acid cycle, is an important mechanism for energy production for the cell and is highly dependent on amino acid availability since amino acids enter this cycle through conversion into a few main TCA cycle intermediates (76). Oxaloacetic acid, ketoglutaric acid, succinyl-CoA and fumaric acid are the main entrance points of the TCA cycle for a selection of few amino acids, whereas other amino acids are linked to the TCA cycle through their conversions into pyruvate and acetyl-CoA, which then enter the TCA cycle at the oxaloacetic acid or citric acid entry point. We analyzed the contents of the metabolites in TCA cycle to obtain results which will serve as an indication of whether the alteration of amino acids happens as a result of protein degradation only, or there are disruptions in the related metabolic cycles, which would change the amino acids consumption, and their involvement in the response to the oxidative stress.

Observing the results of the HaCaT and HEK-293 cell lines we noticed differences in the concentrations of the metabolites involved in TCA cycle, as well as differences in the reaction to the selected treatments. Namely, since HEK-293 have initially lower concentrations of these metabolites, the responses were not as obvious as in HaCaT cells. However, the malic acid was one metabolite with consistent results in both cell lines, and its depletion was more obvious in PtNPs-40 treated cells than in cells treated with PtNPs-10. To understand this variation, we must include oxaloacetic acid Asp, which together they comprise the malate-aspartate shuttle. The malate-aspartate shuttle regulates the concentrations of Asp, oxaloacetic acid, malic acid, which undergo interconversion, a process that is responsible for regulating NAD^+^ levels (77). Both treatments lead the direction of the malic acid-aspartate shuttle equilibrium in favor of oxaloacetic acid, and the increase of Asp upon PtNPs-40 treatment, correlates with the more severe depletion of malic acid. Tully et al. showed that combined drugs such as CDDP and biguanide drugs affect the malate-aspartate shuttle, increasing cytotoxicity by decreasing the concentrations of Asp and NAD^+^ (78).

The decreased concentration of ketoglutaric acid upon PtNPs-10 treatment coincides with the higher oxidative stress, compared to the PtNPs-40 treatment. The increased levels of ROS could cause an increased depletion of ketoglutaric acid, which was shown to have a role in neutralization of ROS (79). This is supported by the higher ketoglutaric acid contents and increased concentrations of Glu and Asp in PtNPs-40 treated HaCaT cells which are the main acids involved in the amino transfer accompanying the reversible interconversion between these ketoglutaric and oxaloacetic acid, catalyzed by aspartate aminotransferase (80).

Most of the TCA metabolites were depleted to provide more energy, however, citric acid in HaCaT cells was increased upon the treatment with PtNPs-10. Incidentally, Ser, the amino acid that is involved in pyruvate production was significantly depleted in both cell lines, which supports the increased production of citric acid. However, it was shown that the abundance of citric acid suppresses glycolysis, and pyruvate dehydrogenase, which impedes the TCA cycle and subsequently induces apoptosis (81).

The participants of the TCA cycle had considerably lower concentrations in HEK-293 cells than in HaCaT cells; thus, we will discuss only the most apparent deviations of the treated cells compared to the control group (**Figure 6**). Regarding HEK-293 treated with PtNPs-10, the decrease of ketoglutaric acid was accompanied an increased concentration of Arg, arising from ketoglutaric acid’s precursor Glu, preferring the conversion of Glu to Arg over ketoglutaric acid. On the other hand, the decrease in ketoglutaric acid caused by PtNPs-40 can be related to the increased concentrations of His and Glu, indicating interference in the deamination reaction of Glu to ketoglutaric acid in the abundant presence of these precursors.

The methionine cycle branches away from homocysteine in the direction of the production of cystathionine and Cys to finally synthesize GSH, an antioxidant enzyme. When there is an increase in oxidative stress, the need for GSH is increased; thus, cystathionine is expected to decrease rapidly to compensate for the GSH requirements. Similar effects to those of cystathionine have been observed in HCT116 cells upon treatment with oxaliplatin (74). The decrease in GSH in HEK-293 cells is an anticipated response resulting from activation of the antioxidant defense mechanism against ROS, where GSH is oxidized in the process of ROS neutralization, increasing the concentration of oxidized glutathione (GSSG). This type of response has been repeatedly observed in cells treated with various cytostatic drugs (CDDP, oxaliplatin, doxorubicin) (74, 82, 83), as well as after PtNP treatment (70, 84, 85). The increased concentration of GSH in HaCaT is atypical in cells subjected to stress caused by PtNPs treatment, and such findings are lacking in related research. However, the fact that GSH was depleted in HEK-293 cells more rapidly than in HaCaT cells showed that HEK-293 cells are more susceptible to oxidative stress, which was also supported by the data obtained from the MTT assay, showing the higher sensitivity of HEK-293 cells to lower concentrations of PTNPs compared to HaCaT cells. Nevertheless, the methionine cycle, apart from Cys and Met, involves Ser as a source for cystathionine synthesis, whereas the subsequent GSH synthesis depends primarily on the availability of Cys, Glu and Gly.

The increase of ketoglutaric acid and GSH, as well as the decease of cystathionine in HaCaT cells treated with PtNPs-40 show that the cells can manage the oxidative stress more easily compared to the cells treated with PtNPs-10, especially since we evidenced that PtNPs-10 cause higher ROS induction. On the other hand, the less apparent increase of GSH, the decrease of ketoglutaric acid, Ser, and cystathionine upon PtNPs-10 treatment, shows that the higher induction od ROS requires a more extreme antioxidant response.

As previously mentioned, all these amino acids serve different purposes and participate in other metabolic cycles, including the TCA cycle, where different amino acids are interconverted into intermediates of the cycle. Thus, for the cells to prevail against oxidative stress, they redirect these metabolic processes and amino acids, prioritizing reaction mechanisms that will promote cell survival and consequently disrupting the balance of the amino acid contents and other participants in the cycles.

### The *ex vivo* effect of platinum-based drugs on the liver and kidneys of chicken embryo

The *in vitro* effects of different PtNP treatments on different cell lines showed contrasting results in the cell lines subjected to the treatments, as well as variability among different amino acids under the selected treatments. This led to the presumption that tissue analyses could show similar variances. The major effect of injection with any platinum drug was a decrease in most of the amino acids in both the liver and kidneys, although the effects were more apparent in the liver than in the kidneys, indicating that the kidneys are less responsive to treatment with platinum drugs than the liver. The amino acid profiles changed most profoundly upon treatment with PtNPs-10, whereas treatment with CDDP and PtNPs-40 displayed slightly less obvious but still significant changes were observed.

In different biological systems presented in different tissues, the composition of biomolecules, electrolytes, pH value varies even with one organ with a unique architectural organization of different cell types (86, 87). Even though the molecular weight of the PVP is the dominant factor determining the release of ions, the NPs interact differently with the compounds they encounter in the different tissues (88). This can additionally affect the rate at which the ions are released, as well the availability of the released ions which can react with the electrolytes found in specific tissue, and the following precipitation of the products can prevent the Pt^2+^ from further action. In both tissues PtNPs-10 showed highest alteration of the amino acid profile, proving that the ion release rate overcomes the effect of the environment. The PtNPs-40 caused a lower effect to the amino acid profile in both tissues, however, in kidneys this effect was comparable with CDDP. The possible explanation is that the effect of the surrounding compounds in the different tissues is more obvious with PtNPs-40 than PtNPs-10, and due to the slower rate of ion release, the PtNPs-40 cannot compensate for the interactions with the compounds found in their proximity. Moreover, it’s shown that the molecular weight of some polymers determines the distribution to different organs (68).

Both tissues showed a general decrease in amino acid concentrations. In agreement with our results is the similar observation of the behavior of most of the amino acids reported in myocardial tissue of chicken after the treatment with doxorubicin (39). Several studies on serum analyses in rats also reported alterations in amino acid profiles. Namely, CDDP caused a decrease in BCAAs (89), whereas doxorubicin decreased the amounts of Thr, Met, Arg, Phe, Ala, Leu and Val in the analyzed sera of rats (90). On the other hand, low glucose levels were detected in the blood of mice treated with PEG-PtNPs, diminishing the main energy source needed for growth and cell survival (91). Alternative energy sources include non-glucose compounds such as amino acids, which can alleviate energy deficiency through gluconeogenesis, being depleted themselves, as well as protein breakdown providing free amino acids to support gluconeogenesis (90). A corresponding finding of our study is the significantly diminished weight of our chicken embryos after treatment with any platinum form compared with the control; however, no differences were found between the weight of embryos subjected to the different treatments (**Table S2**). This could indicate that the utilization of amino acids for growth was suppressed to prioritize the survival of the organism. Previous studies showed that treatment with PtNPs at lower concentrations (1-20 μg/mL) did not affect the embryo weight; however, an upper limit of 100 μg/mL was considered a safe dose (92). In our study, two of the concentrations we applied (150 and 300 μg/mL) were above the suggested safe limit, explaining the weight drop upon the given treatments. A pro-survival attempt was revealed in cochlear rat tissue, where treatment with CDDP induced increases in a number of cell-signaling and stress-response proteins that participate in the cell survival mechanism (93). A different explanation was given in several studies reporting increased excretion of amino acids in urine after exposure to different chemotherapeutic agents, implying interference with the tubular reabsorption of amino acids in the kidney (94–97). However, examination of amino acid levels in urine obtained after treatment with PtNPs is lacking.

## Conclusion

CDDP is one of the most conventional cytostatic drugs used as a treatment in various types of cancers; however, many side effects, including overdose-related toxicity, diminish its success rate and limit its application. On the other hand, PtNPs have been suggested as a replacement therapy with excellent anticancer and therapeutic properties. In this study, we investigated the cytotoxic effects of PtNPs-10 and PtNPs-40 on two cell lines, HEK-293 and HaCaT. We established their biocompatibility and examined their impact on amino acid metabolism, as well as the related changes in the TCA and methionine cycles. Furthermore, we used chicken embryos as an *in vivo* model to examine the effects on the amino acid profiles in the liver and kidneys, which are the main tissue locations of amino acid metabolism. HEK-293 cells displayed a higher sensitivity to the PtNPs than to HaCaT cells, whereas CDDP was much more toxic to HaCaT cells than PtNPs. The PtNPs were successfully internalized by the cells, where they can employ their mechanism of action and potential toxicity without causing damage to the membrane or DNA. The oxidative stress caused by PtNP treatments activated the antioxidant defense mechanism, causing changes in the concentration of GSH. The related amino acids included in the synthesis of GSH (Cys, Glu and Gly) and in the methionine cycle (Met and Ser) underwent changes in their concentrations in the cells, causing an imbalance in the overall amino acid metabolism, consequently affecting the metabolic transformations of the metabolites participating in the TCA cycle. In the liver and kidneys of chicken embryos treated with PtNPs, a general decrease in the concentration of amino acids occurred, which was more evident in the liver than in the kidney. The dissipation of the amino acids was accompanied by a decrease in the weight of the treated embryos. Treatment with PtNPs-10 was associated with the most prominent changes in amino acids in both tissues, whereas PtNPs-40 affected the concentration of only a few amino acids. However, since PtNPs-10 treatment affected the amino acid profile of the liver and kidneys more than conventional treatment with CDDP, the results raise concerns regarding whether this treatment provides enough benefits as cytostatic to outweigh its side effects. On the other hand, treatment with PtNPs-40 displayed the lowest alteration of the amino acid profile in the liver and kidneys, presenting a promising alternative for the existing platinum treatments.

## Data availability statement

The original contributions presented in the study are included in the article/**Supplementary Material**. Further inquiries can be directed to the corresponding author.

## Ethics statement

Ethical review and approval was not required for the animal study because All Institutional and National Guidelines for the care and use of animals (fisheries) were followed. In the European Union countries, CAM assay is not considered an animal experiment and, therefore, it does not require ethical approval.

## Author contributions

KM: Investigation, Validation, Formal analysis, Visualization, Writing - Original Draft; NC: Methodology, Investigation, Validation; HM: Investigation, Validation, Data curation; MR: Validation, Writing – review & editing; LS: Investigation, Validation, Formal analysis; ZH: Writing – review & editing; OZ: Writing – review & editing; PK: Investigation, Validation, Visualization; VA: Resources, Writing – review & editing, Project administration, Funding acquisition; VM: Conceptualization, Supervision, Visualization, Writing - Review & Editing, Data curation. All authors contributed to the article and approved the submitted version.

## Funding

This research was carried out under the project CEITEC 2020 (LQ1601) with financial support from the Ministry of Education, Youth and Sports of the Czech Republic under the National Sustainability Programme II.

## Conflict of interest

The authors declare that the research was conducted in the absence of any commercial or financial relationships that could be construed as a potential conflict of interest.

## Publisher’s note

All claims expressed in this article are solely those of the authors and do not necessarily represent those of their affiliated organizations, or those of the publisher, the editors and the reviewers. Any product that may be evaluated in this article, or claim that may be made by its manufacturer, is not guaranteed or endorsed by the publisher.

## References

[1] Dasari S Tchounwou PB. Cisplatin in cancer therapy: Molecular mechanisms of action. Eur J Pharmacol (2014) 0:364–78. doi: 10.1016/j.ejphar.2014.07.025 PMC4146684 25058905

[2] Avan A Postma TJ Ceresa C Cavaletti G Giovannetti E Peters GJ. Platinum-induced neurotoxicity and preventive strategies: Past, present, and future. Oncologist (2015) 20(4):411–32. doi: 10.1634/theoncologist.2014-0044 PMC4391771 25765877

[3] Bosl GJ Motzer RJ. Testicular germ-cell cancer. N Engl J Med (1997) 337(4):242–53. doi: 10.1056/NEJM199707243370406 9227931

[4] Heger Z Gumulec J Cernei N Tmejova K Kopel P Balvan J. 17beta-estradiol-containing liposomes as a novel delivery system for the antisense therapy of ER-positive breast cancer: An *in vitro* study on the MCF-7 cell line. Oncol Rep (2015) 33(2):921–9. doi: 10.3892/or.2014.3627 25434399

[5] Oun R Rowan E. Cisplatin induced arrhythmia; electrolyte imbalance or disturbance of the SA node? Eur J Pharmacol (2017) 811:125–8. doi: 10.1016/j.ejphar.2017.05.063 28599874

[6] Starobova H Vetter I. Pathophysiology of chemotherapy-induced peripheral neuropathy. Front Mol Neurosci (2017) 10:174. doi: 10.3389/fnmol.2017.00174 28620280 PMC5450696

[7] Langevin S Chang JS Chang S. Serious retinopathy associated with cisplatin treatment. RETIn. Cases Brief Rep (2019) 13(3):211–4. doi: 10.1097/ICB.0000000000000573 28333855

[8] Tsuji D Suzuki K Kawasaki Y Goto K Matsui R Seki N. Risk factors associated with chemotherapy-induced nausea and vomiting in the triplet antiemetic regimen including palonosetron or granisetron for cisplatin-based chemotherapy: Analysis of a randomized, double-blind controlled trial. Support Care Cancer (2019) 27(3):1139–47. doi: 10.1007/s00520-018-4403-y 30094732

[9] Hwang DB Won DH Shin YS Kim SY Kang BC Lim KM. Ccrn4l as a pre-dose marker for prediction of cisplatin-induced hepatotoxicity susceptibility. Free Radic Biol Med (2020) 148:128–39. doi: 10.1016/j.freeradbiomed.2020.01.003 31911150

[10] Manyau PMC Mabeka M Mudzviti T Kadzatsa W Nyamhunga A. Renal function impairment in cervical cancer patients treated with cisplatin-based chemoradiation: A review of medical records in a Zimbabwean outpatient department. PloS One (2021) 16(2):e0245383. doi: 10.1371/journal.pone.0245383 33626044 PMC7904141

[11] Amptoulach S Tsavaris N. Neurotoxicity caused by the treatment with platinum analogues. Chemother. Res Pract (2011), 3, 843019. doi: 10.1155/2011/843019 PMC3265255 22312559

[12] Jurek T Rorat M Dys P Swiatek B. Fatal cisplatin overdose in the treatment of mediastinal lymphoma with the ESHAP regimen - analysis of the causes of the adverse drug event. Onkologie (2013) 36(1-2):49–52. doi: 10.1159/000346677 23429332

[13] Bowden NA. Nucleotide excision repair: Why is it not used to predict response to platinum-based chemotherapy? Cancer Lett (2014) 346(2):163–71. doi: 10.1016/j.canlet.2014.01.005 24462818

[14] He CB Lu KD Liu DM Lin WB. Nanoscale metal-organic frameworks for the Co-delivery of cisplatin and pooled siRNAs to enhance therapeutic efficacy in drug-resistant ovarian cancer cells. J Am Chem Soc (2014) 136(14):5181–4. doi: 10.1021/ja4098862 PMC4210117 24669930

[15] Kim YK Jung JS Lee SH Kim YW. Effects of antioxidants and Ca2+ in cisplatin-induced cell injury in rabbit renal cortical slices. Toxicol Appl Pharmacol (1997) 146(2):261–9. doi: 10.1006/taap.1997.8252 9344894

[16] Panesso MC Shi MJ Cho HJ Paek J Ye JF Moe OW. Klotho has dual protective effects on cisplatin-induced acute kidney injury. Kidney Int (2014) 85(4):855–70. doi: 10.1038/ki.2013.489 PMC3972320 24304882

[17] Cersosimo RJ. Hepatotoxicity associated with cisplatin chemotherapy. Ann Pharmacother (1993) 27(4):438–41. doi: 10.1177/106002809302700408 8477119

[18] Lu YK Cederbaum AI. Cisplatin-induced hepatotoxicity is enhanced by elevated expression of cytochrome P450 2E1. Toxicol. Sci (2006) 89(2):515–23. doi: 10.1093/toxsci/kfj031 16251482

[19] Cavalli F Tschopp L Sonntag RW Zimmermann A. Case of liver toxicity following cis- dichlorodiammineplatinum(ll)treatment. Cancer Treat Rep (1978) 62(12):2125–6. 751721

[20] Dasari S Tchounwou PB. Cisplatin in cancer therapy: Molecular mechanisms of action. Eur J Pharmacol (2014) 740:364–78. doi: 10.1016/j.ejphar.2014.07.025 PMC4146684 25058905

[21] Koc A Duru M Ciralik H Akcan R Sogut S. Protective agent, erdosteine, against cisplatin-induced hepatic oxidant injury in rats. Mol Cell Biochem (2005) 278(1-2):79–84. doi: 10.1007/s11010-005-6630-z 16180092

[22] Olszewski U Ulsperger E Geissler K Hamilton G. Comparison of the effects of the oral anticancer platinum(IV) complexes oxoplatin and metabolite cis-diammine-tetrachlorido-platinum(IV) on global gene expression of NCI-H526 cells. J Exp Pharmacol (2011) 3:43–50. doi: 10.2147/JEP.S13630 27186109 PMC4863305

[23] Hauert S Bhatia SN. Mechanisms of cooperation in cancer nanomedicine: Towards systems nanotechnology. Trends Biotechnol (2014) 32(9):448–55. doi: 10.1016/j.tibtech.2014.06.010 PMC4295824 25086728

[24] Cao GJ Fisher CM Jiang XM Chong Y Zhang H Guo HY. Platinum nanoparticles: an avenue for enhancing the release of nitric oxide from s-nitroso-N-acetylpenicillamine and s-nitrosoglutathione. Nanoscale (2018) 10(23):11176–85. doi: 10.1039/c8nr03874k 29873378

[25] Ma Y Wang ZH Ma YX Han ZH Zhang M Chen HY. A telomerase-responsive DNA icosahedron for precise delivery of platinum nanodrugs to cisplatin-resistant cancer. Angewandte Chemie-Int Edition (2018) 57(19):5389–93. doi: 10.1002/anie.201801195 29569826

[26] Pajic MNK Stevanovic SI Radmilovic VV Gavrilovic-Wohlmuther A Radmilovic VR Gojkovic SL. Shape evolution of carbon supported pt nanoparticles: From synthesis to application. Appl Catal B-Environ (2016) 196:174–84. doi: 10.1016/j.apcatb.2016.05.033

[27] Li L Wang CP Huang Q Xiao JR Zhang Q Cheng YY. And oxidized dextran for repeated photothermal cancer therapy. J Mater Chem B (2018) 6(16):2474–80. doi: 10.1039/c8tb00091c 32254464

[28] Rezaei SJT Hesami A Khorramabadi H Amani V Malekzadeh AM Ramazani A. Pt(II) complexes immobilized on polymer-modified magnetic carbon nanotubes as a new platinum drug delivery system. Appl Organomet Chem (2018) 32(7):e4401. doi: 10.1002/aoc.4401

[29] Horie M Kato H Endoh S Fujita K Nishio K Komaba LK. Evaluation of cellular influences of platinum nanoparticles by stable medium dispersion. Metallomics (2011) 3(11):1244–52. doi: 10.1039/c1mt00060h 21804981

[30] Asharani PV Xinyi N Hande MP Valiyaveettil S. And p53-mediated growth arrest in human cells treated with platinum nanoparticles. Nanomedicine (2010) 5(1):51–64. doi: 10.2217/nnm.09.85 20025464

[31] Johnstone TC Suntharalingam K Lippard SJ. The next generation of platinum drugs: Targeted Pt(II) agents, nanoparticle delivery, and Pt(IV) prodrugs. Chem Rev (2016) 116(5):3436–86. doi: 10.1021/acs.chemrev.5b00597 PMC4792284 26865551

[32] Nejdl L Kudr J Moulick A Hegerova D Ruttkay-Nedecky B Gumulec J. Platinum nanoparticles induce damage to DNA and inhibit DNA replication. PloS One (2017) 12(7):e0180798. doi: 10.1371/journal.pone.0180798 28704436 PMC5507526

[33] Sikder M Wang J Chandler GT Berti D Baalousha M. Synthesis, characterization, and environmental behaviors of monodispersed platinum nanoparticles. J Colloid Interface Sci (2019) 540:330–41. doi: 10.1016/j.jcis.2019.01.036 30660085

[34] Sorensen SN Engelbrekt C Lutzhoft HH Jimenez-Lamana J Noori JS Alatraktchi FA. A multimethod approach for investigating algal toxicity of platinum nanoparticles. Environ Sci Technol (2016) 50(19):10635–43. doi: 10.1021/acs.est.6b01072 27577171

[35] Lin CX Gu JL Cao JM. The acute toxic effects of platinum nanoparticles on ion channels, transmembrane potentials of cardiomyocytes *in vitro* and heart rhythm *in vivo* in mice. Int J Nanomed. (2019) 14:5595–609. doi: 10.2147/IJN.S209135 PMC6660630 31413565

[36] Katao K Honma R Kato S Watanabe S Imai J. Influence of platinum nanoparticles orally administered to rats evaluated by systemic gene expression profiling. Exp Anim (2011) 60(1):33–45. doi: 10.1538/expanim.60.33 21325750

[37] Eliezar J Scarano W Boase NRB Thurecht KJ Stenzel MH. *In vivo* evaluation of folate decorated cross-linked micelles for the delivery of platinum anticancer drugs. Biomacromolecules (2015) 16(2):515–23. doi: 10.1021/bm501558d 25543837

[38] Liu L Ye Q Lu MG Lo YC Hsu YH Wei MC. A new approach to reduce toxicities and to improve bioavailabilities of platinum-containing anti-cancer nanodrugs. Sci Rep (2015) 5:10881. doi: 10.1038/srep10881 26039249 PMC4454134

[39] Heger Z Cernei N Kudr J Gumulec J Blazkova I Zitka O. A novel insight into the cardiotoxicity of antineoplastic drug doxorubicin. Int J Mol Sci (2013) 14(11):21629–46. doi: 10.3390/ijms141121629 PMC3856025 24185911

[40] Vieiramakings E Vanderwesthuyzen J Metz J. Both valine and isoleucine supplementation delay the development of neurological impairment in vitamin b-12 deficient bats. Int J Vitam. Nutr Res (1990) 60(1):41–6. 2387670

[41] Harper AE Miller RH Block KP. Branched -chain amino-acid -metabolism. Annu Rev Nutr (1984) 4:409–54. doi: 10.1146/annurev.nu.04.070184.002205 6380539

[42] Holdsworth JD Dionigi P Clague MB James OFW Wright PD. Body protein -metabolism and plasma amino-acids in cirrhosis of the liver-the effect of varying the branched-chain amino-acid content of intravenous amino-acid solutions. Clin Nutr (1984) 3(3):153–62. doi: 10.1016/0261-5614(84)90048-7 16829451

[43] Kosanam H Thai K Zhang Y Advani A Connelly KA Diamandis EP. Diabetes induces lysine acetylation of intermediary metabolism enzymes in the kidney. Diabetes (2014) 63(7):2432–9. doi: 10.2337/db12-1770 24677711

[44] Buchtelova H Dostalova S Michalek P Krizkova S Strmiska V Kopel P. Size-related cytotoxicological aspects of polyvinylpyrrolidone-capped platinum nanoparticles. Food Chem Toxicol (2017) 105:337–46. doi: 10.1016/j.fct.2017.04.043 28465190

[45] Long NV Ohtaki M Nogami M Hien TD. Effects of heat treatment and poly(vinylpyrrolidone) (PVP) polymer on electrocatalytic activity of polyhedral pt nanoparticles towards their methanol oxidation. Colloid Polym. Sci (2011) 289(12):1373–86. doi: 10.1007/s00396-011-2460-6

[46] Borowik A Banasiuk R Derewonko N Rychlowski M Krychowiak-Masnicka M Wyrzykowski D. Interactions of newly synthesized platinum nanoparticles with ICR-191 and their potential application. Sci Rep (2019) 9(1):4987. doi: 10.1038/s41598-019-41092-6 30899037 PMC6428851

[47] Teow Y Valiyaveettil S. Active targeting of cancer cells using folic acid-conjugated platinum nanoparticles. Nanoscale (2010) 2(12):2607–13. doi: 10.1039/c0nr00204f 20936240

[48] Yue B Ma Y Tao H Yu L Jian G Wang X. CN x nanotubes as catalyst support to immobilize platinum nanoparticles for methanol oxidation. J Mater. Chem (2008) 18(15):1747–50. doi: 10.1039/b718283j

[49] Mazzotta E Rella S Turco A Malitesta C. XPS in development of chemical sensors. RSC Adv (2015) 5(101):83164–86. doi: 10.1039/C5RA14139G

[50] Mondal A Jana NR. Effect of size and oxidation state of platinum nanoparticles on the electrocatalytic performance of graphene-nanoparticle composites. RSC Adv (2015) 5(104):85196–201. doi: 10.1039/C5RA17087G

[51] Moulder JF Stickle WF Sobol PE Bomben KD. Handbook of X Ray Photoelectron Spectroscopy. A Reference Book of Standard Spectra for Identification and Interpretation. Muilenberg GE, editor. (Norwalk: Physical Electronics Division, Perkin-Elmer Corp) (1995).

[52] Tian ZQ Jiang SP Liang YM Shen PK. Synthesis and characterization of platinum catalysts on multiwalled carbon nanotubes by intermittent microwave irradiation for fuel cell applications. J Phys Chem B (2006) 110(11):5343–50. doi: 10.1021/jp056401o 16539467

[53] Villers D Sun S Serventi A Dodelet J Desilets S. Characterization of pt nanoparticles deposited onto carbon nanotubes grown on carbon paper and evaluation of this electrode for the reduction of oxygen. J Phys Chem B (2006) 110(51):25916–25. doi: 10.1021/jp065923g 17181240

[54] Borodko Y Humphrey SM Tilley TD Frei H Somorjai GA. Charge-transfer interaction of poly(vinylpyrrolidone) with platinum and rhodium nanoparticles. J Phys Chem C (2007) 111(17):6288–95. doi: 10.1021/jp068742n

[55] Borodko Y Habas SE Koebel M Yang P Frei H Somorjai GA. Probing the interaction of poly (vinylpyrrolidone) with platinum nanocrystals by UV– raman and FTIR. J Phys Chem B (2006) 110(46):23052–9. doi: 10.1021/jp063338+ 17107143

[56] Ye J-Y Attard GA Brew A Zhou Z-Y Sun S-G Morgan DJ. Explicit detection of the mechanism of platinum nanoparticle shape control by polyvinylpyrrolidone. J Phys Chem C (2016) 120(14):7532–42. doi: 10.1021/acs.jpcc.5b10910

[57] Louie SM Gorham JM Tan J Hackley VA. Ultraviolet photo-oxidation of polyvinylpyrrolidone (PVP) coatings on gold nanoparticles. Environ Sci.: Nano (2017) 4(9):1866–75. doi: 10.1039/C7EN00411G PMC6508591 31080619

[58] Burger H Zoumaro-Djayoon A Boersma A Helleman J Berns E Mathijssen R. Differential transport of platinum compounds by the human organic cation transporter hOCT2 (hSLC22A2). Br J Pharmacol (2010) 159(4):898–908. doi: 10.1111/j.1476-5381.2009.00569.x 20067471 PMC2829215

[59] Murin R Vidomanova E Kowtharapu BS Hatok J Dobrota D. Role of s-adenosylmethionine cycle in carcinogenesis. Gen Physiol Biophys (2017) 36(5):513–20. doi: 10.4149/gpb_2017031 29372684

[60] Masood F. Polymeric nanoparticles for targeted drug delivery system for cancer therapy. Mater Sci Eng: C (2016) 60:569–78. doi: 10.1016/j.msec.2015.11.067 26706565

[61] Zhang X Zeng G Tian J Wan Q Huang Q Wang K. PEGylation of carbon nanotubes *via* mussel inspired chemistry: Preparation, characterization and biocompatibility evaluation. Appl Surf Sci (2015) 351:425–32. doi: 10.1016/j.apsusc.2015.05.160

[62] Caracciolo G Farokhzad OC Mahmoudi M. Biological identity of nanoparticles *in vivo*: clinical implications of the protein corona. Trends Biotechnol (2017) 35(3):257–64. doi: 10.1016/j.tibtech.2016.08.011 27663778

[63] Li Y Deng Y Tian X Ke H Guo M Zhu A. Multipronged design of light-triggered nanoparticles to overcome cisplatin resistance for efficient ablation of resistant tumor. ACS Nano (2015) 9(10):9626–37. doi: 10.1021/acsnano.5b05097 26365698

[64] Pedone D Moglianetti M De Luca E Bardi G Pompa P.P.J.C.S.R. Platinum nanoparticles in nanobiomedicine. Chemical Society Reviews (2017) 46(16):4951–75. doi: 10.1039/C7CS00152E 28696452

[65] Perioli L Ambrogi V Angelici F Ricci M Giovagnoli S Capuccella M. Development of mucoadhesive patches for buccal administration of ibuprofen. J Control Release (2004) 99(1):73–82. doi: 10.1016/j.jconrel.2004.06.005 15342182

[66] Diaz del Consuelo I Falson F Guy RH Jacques Y. *Ex vivo* evaluation of bioadhesive films for buccal delivery of fentanyl. J Control Release (2007) 122(2):135–40. doi: 10.1016/j.jconrel.2007.05.017 17688966

[67] Rasekh M Karavasili C Soong YL Bouropoulos N Morris M Armitage D. Electrospun PVP-indomethacin constituents for transdermal dressings and drug delivery devices. Int J Pharm (2014) 473(1-2):95–104. doi: 10.1016/j.ijpharm.2014.06.059 24997411

[68] Kaneda Y Tsutsumi Y Yoshioka Y Kamada H Yamamoto Y Kodaira H. The use of PVP as a polymeric carrier to improve the plasma half-life of drugs. Biomaterials (2004) 25(16):3259–66. doi: 10.1016/j.biomaterials.2003.10.003 14980420

[69] Gatto F Moglianetti M Pompa PP Bardi G. Platinum nanoparticles decrease reactive oxygen species and modulate gene expression without alteration of immune responses in THP-1 monocytes. Nanomater. (Basel) (2018) 8(6):392. doi: 10.3390/nano8060392 PMC6027382 29865145

[70] Almarzoug MHA Ali D Alarifi S Alkahtani S Alhadheq AM. Platinum nanoparticles induced genotoxicity and apoptotic activity in human normal and cancer hepatic cells *via* oxidative stress-mediated Bax/Bcl-2 and caspase-3 expression. Environ Toxicol (2020) 35(9):930–41. doi: 10.1002/tox.22929 32309901

[71] Franco P De Marco I. The use of Poly(N-vinyl pyrrolidone) in the delivery of drugs: A review. Polym. (Basel) (2020) 12(5):1114. doi: 10.3390/polym12051114 PMC7285361 32414187

[72] Jan H Gul R Andleeb A Ullah S Shah M Khanum M. A detailed review on biosynthesis of platinum nanoparticles (PtNPs), their potential antimicrobial and biomedical applications. J Saudi Chem Soc (2021) 25(8):101297. doi: 10.1016/j.jscs.2021.101297

[73] Kim HR Park JH Lee SH Kwack SJ Lee J Kim S. Using intracellular metabolic profiling to identify novel biomarkers of cisplatin-induced acute kidney injury in NRK-52E cells. J Toxicol Environ Health A (2022) 85(1):29–42. doi: 10.1080/15287394.2021.1969305 34445936

[74] Galvez L Rusz M Schwaiger-Haber M El Abiead Y Hermann G Jungwirth U. Preclinical studies on metal based anticancer drugs as enabled by integrated metallomics and metabolomics. Metallomics (2019) 11(10):1716–28. doi: 10.1039/c9mt00141g 31497817

[75] Gunda V Pathania AS Chava S Prathipati P Chaturvedi NK Coulter DW. Amino acids regulate cisplatin insensitivity in neuroblastoma. Cancers (Basel) (2020) 12(9):2576. doi: 10.3390/cancers12092576 PMC7563727 32927667

[76] Pasini E Corsetti G Aquilani R Romano C Picca A Calvani R. Protein-amino acid metabolism disarrangements: The hidden enemy of chronic age-related conditions. Nutrients (2018) 10(4):391. doi: 10.3390/nu10040391 PMC5946176 29565819

[77] Borst P. The malate-aspartate shuttle (Borst cycle): How it started and developed into a major metabolic pathway. IUBMB Life (2020) 72(11):2241–59. doi: 10.1002/iub.2367 PMC7693074 32916028

[78] Tully E Bharti S Woo J Bhujwalla Z Gabrielson E. Biguanide drugs enhance cytotoxic effects of cisplatin by depleting aspartate and NAD+ in sensitive cancer cells. Cancer Biol Ther (2021) 22(10-12):579–86. doi: 10.1080/15384047.2021.1982599 PMC8726685 34720054

[79] Mailloux RJ Beriault R Lemire J Singh R Chenier DR Hamel RD. The tricarboxylic acid cycle, an ancient metabolic network with a novel twist. PloS One (2007) 2(8):e690. doi: 10.1371/journal.pone.0000690 17668068 PMC1930152

[80] Ozaki T Ishiguro S Itoh H Furuhama K Nakazawa M Yamashita T. Cisplatin binding and inactivation of mitochondrial glutamate oxaloacetate transaminase in cisplatin-induced rat nephrotoxicity. Biosci Biotechnol Biochem (2013) 77(8):1645–9. doi: 10.1271/bbb.130172 23924727

[81] Ren JG Seth P Ye H Guo K Hanai JI Husain Z. Citrate suppresses tumor growth in multiple models through inhibition of glycolysis, the tricarboxylic acid cycle and the IGF-1R pathway. Sci Rep (2017) 7(1):4537. doi: 10.1038/s41598-017-04626-4 28674429 PMC5495754

[82] Ryu CS Kwak HC Lee KS Kang KW Oh SJ Lee KH. Sulfur amino acid metabolism in doxorubicin-resistant breast cancer cells. Toxicol Appl Pharmacol (2011) 255(1):94–102. doi: 10.1016/j.taap.2011.06.004 21703291

[83] Ali M Manjula SN Wani SUD Parihar VK Mruthunjaya KM Madhunapantula SV. Protective role of herbal formulation-divine noni against cisplatin-induced cytotoxicity in healthy cells by activating Nrf2 expression: An *in-vivo* and *in-vitro* approach. Phytomed. Plus (2021) 1(1):100009. doi: 10.1016/j.phyplu.2020.100009

[84] Gurunathan S Jeyaraj M Kang MH Kim JH. The effects of apigenin-biosynthesized ultra-small platinum nanoparticles on the human monocytic THP-1 cell line. Cells (2019) 8(5):444. doi: 10.3390/cells8050444 PMC6562931 31083475

[85] Gurunathan S Jeyaraj M La H Yoo H Choi Y Do JT. Anisotropic platinum nanoparticle-induced cytotoxicity, apoptosis, inflammatory response, and transcriptomic and molecular pathways in human acute monocytic leukemia cells. Int J Mol Sci (2020) 21(2):440. doi: 10.3390/ijms21020440 PMC7014054 31936679

[86] Du B Yu M Zheng J. Transport and interactions of nanoparticles in the kidneys. Nat Rev Mater (2018) 3(10):358–74. doi: 10.1038/s41578-018-0038-3

[87] Zhang YN Poon W Tavares AJ McGilvray ID Chan WCW. Nanoparticle-liver interactions: Cellular uptake and hepatobiliary elimination. J Control Release (2016) 240:332–48. doi: 10.1016/j.jconrel.2016.01.020 26774224

[88] Ronavari A Belteky P Boka E Zakupszky D Igaz N Szerencses B. Polyvinyl-Pyrrolidone-Coated silver nanoparticles-the colloidal, chemical, and biological consequences of steric stabilization under biorelevant conditions. Int J Mol Sci (2021) 22(16):8673. doi: 10.3390/ijms22168673 34445378 PMC8395525

[89] Zhang P Li W Chen J Li R Zhang Z Huang Y. Branched-chain amino acids as predictors for individual differences of cisplatin nephrotoxicity in rats: A pharmacometabonomics study. J Proteome Res (2017) 16(4):1753–62. doi: 10.1021/acs.jproteome.7b00014 28271897

[90] Forsgard RA Marrachelli VG Korpela K Frias R Collado MC Korpela R. Chemotherapy-induced gastrointestinal toxicity is associated with changes in serum and urine metabolome and fecal microbiota in male sprague-dawley rats. Cancer Chemother Pharmacol (2017) 80(2):317–32. doi: 10.1007/s00280-017-3364-z PMC5532424 28646338

[91] Mukherjee S Bollu VS Roy A Nethi SK Madhusudana K Kumar JM. Acute toxicity, biodistribution, and pharmacokinetics studies of pegylated platinum nanoparticles in mouse model. Adv. NanoBiomed Res (2021) 1(7):2000082. doi: 10.1002/anbr.202000082

[92] Prasek M Sawosz E Jaworski S Grodzik M Ostaszewska T Kamaszewski M. Influence of nanoparticles of platinum on chicken embryo development and brain morphology. Nanoscale Res. Lett (2013) 8(1):251–9. doi: 10.1186/1556-276X-8-251 PMC3664603 23705751

[93] Coling DE Ding D Young R Lis M Stofko E Blumenthal KM. Proteomic analysis of cisplatin-induced cochlear damage: Methods and early changes in protein expression. Hear Res (2007) 226(1-2):140–56. doi: 10.1016/j.heares.2006.12.017 17321087

[94] Portilla D Li S Nagothu KK Megyesi J Kaissling B Schnackenberg L. Metabolomic study of cisplatin-induced nephrotoxicity. Kidney Int (2006) 69(12):2194–204. doi: 10.1038/sj.ki.5000433 16672910

[95] Gamelin L Capitain O Morel A Dumont A Traore S Anne le B. Predictive factors of oxaliplatin neurotoxicity: The involvement of the oxalate outcome pathway. Clin Cancer Res (2007) 13(21):6359–68. doi: 10.1158/1078-0432.CCR-07-0660 17975148

[96] Portilla D Schnackenberg L Beger RD. Metabolomics as an extension of proteomic analysis: study of acute kidney injury. Semin Nephrol (2007) 27(6):609–20. doi: 10.1016/j.semnephrol.2007.09.006 PMC2684501 18061843

[97] Pariyani R Ismail IS Azam A Khatib A Abas F Shaari K. Urinary metabolic profiling of cisplatin nephrotoxicity and nephroprotective effects of orthosiphon stamineus leaves elucidated by (1)H NMR spectroscopy. J Pharm BioMed Anal (2017) 135:20–30. doi: 10.1016/j.jpba.2016.12.010 27987392

