# Supplementary material for: Platinum-based drug-induced depletion of amino acids in the kidneys and liver
Source: Front Oncol. 2022 Sep 21;12:986045. doi: 10.3389/fonc.2022.986045 (PMC9535364; doi:10.3389/fonc.2022.986045)
Supplement: Supplementary file 1 [file DataSheet_1.docx]

**Supporting Information**

**Materials and Methods**

Materials

All chemicals in ACS purity were supplied by Sigma Aldrich (St. Louis, MO, USA).

Synthesis of PVP-capped PtNPs (PtNPs-10 and PtNPs-40)

The platinum nanoparticles were produced by using protocol published in our previous work (Buchtelova et al., 2017). Briefly, 0.07 g of PtCl_4_ (Mr = 336.89) with addition of 33 μL of 37% HCl, was dissolved in 10 mL of water, followed by an addition of 0.14 g of polyvinylpyrrolidone with different molecular weight PVP-10k and PVP-40k. Then, 5 mL of H_2_[PtCl_6_] was added and the mixture was stirred for 1 h. Finally, reduction was achieved by adding 50 mg of Na[BH_4_], and filled up to 50 mL, keeping the solution for 2 h under stirring. Prior the experiments, the content of platinum present in PtNPs solutions were determine by using atomic absorption spectrometer 280Z with Zeeman background correction (Agilent Technologies, Santa Clara, CA, USA).

Characterization of Platinum Nanoparticles (PtNPs-10 and PtNPs-40)

In our preliminary study, we conducted more detailed characterization of PtNPs used in current study (Buchtelova et al., 2017). Despite that, we run some elementary tests to confirm presence and stability of PtNPs in solution. The PtNPs were photographed using Low Voltage Electron Microscope in TEM mode (LVEM5 Delong Instruments, Brno, Czech Republic). Samples for TEM were prepared simple by sedimentation of NPs from a drop of dispersion in water on the microscopic copper TEM grid with carbon layer.. The average particle size and size distribution were determined by particles size analyzer (NANO-ZS, Malvern Instruments Ltd., Worcestershire, U.K.), measured at a detector angle of 173°, a wavelength of 633 nm, a real refractive index of 1.33, and a temperature of 25 °C.

Cell lines and culture conditions

Two different human cell lines were used in this study: i) HaCaT - spontaneously transformed aneuploidy immortal, non-tumorigenic keratinocyte cell line derived from adult human skin, and; ii) HEK-293 – human epithelial cell line derived from kidney tissue. The cell lines used in this study were purchased from the American Type Culture Collection (ATCC, Manassas, VA, USA). The cells were cultured in DMEM with 10% foetal bovine serum (FBS). Media were supplemented with penicillin (100 U/mL) and streptomycin (0.1 mg/mL) and the cells were maintained at 37 °C in a humidified incubator Galaxy^®^ 170 R (Eppendorf, Hamburg, Germany) (Buchtelova et al., 2017).

Viability and proliferation assays

The viability of the cells was assessed applying the MTT (3-(4,5-dimethylthiazol-2-yl)-2,5-diphenyltetrazolium bromide) assay. A suspension of 5000 cells in 50 μL medium was added to each well of microtiter plates, incubating them for 24 h at 37 °C with 5% CO_2_ to ensure cell growth. To effects on cell viability were determined for PtNPs-10, PtNPs-40 and CDDP within concentration range of 0.01-50 μM. Treatment was carried out for 24 h and 48 h. Then, 10 μL of MTT [5 mg/mL in phosphate buffered saline (PBS)] was added to the cells and the mixture was incubated for 4 h at 37 °C. Next, the MTT-containing medium was replaced with 100 μL of 99.9% dimethyl sulfoxide (DMSO) and the absorbance of the samples after 5 min incubation was determined at 570 nm using Infinite 200 PRO (Tecan, Männedorf, Switzerland) (Heger et al., 2015).

*Haemocompatibility*

The haemocompatibility of PtNPs was determined using human red blood cells (RBCs). After collection of the blood by antecubital venipuncture of healthy donor with signed informed consent, RBCs were obtained according to Evans et al (Evans et al., 2013). The RBCs suspensions were washed with 150 mM NaCl solution three-to-five times. Then, four different concentrations of PtNPs (6.2, 12.5, 25.0 and 50.0 μg/mL), diluted in PBS were mixed with RBCs and incubated for 1 h at 37 *°*C. The degree of haemolysis was assessed by measuring the supernatant absorbance at 540 nm after centrifugation and it was calculated according to the following equation:

% haemolysis = [(A_t_ - A_c_)/A_100%_ -A_c_] × 100

where A_t_ is the absorbance of the supernatant from the samples incubated with the PtNPs; A_c_ is the absorbance of the supernatant from the negative control (PBS, pH 7.4); A_100%_ is the absorbance of the supernatant of positive control (0.1% Triton X-100), causing complete lysis of RBCs.

*PtNPs internalization and cell morphology visualization*

CLSM (LSM 880, Carl Zeiss, Jena, Germany) was used to visualize PtNPs by AiryScan imaging. The cells were stained according to manufacturer’s protocol with Alexa Fluor 488 Dye (Thermo Fisher Scientific) to stain F-actin. Nuclei were counterstained with Hoechst 33258. To visualize the NPs, the cells were excited at 633 nm laser with emissions at 625-637 nm. The visualization of the Hoechst stained nuclei was achieved at 405 nm excitation and 445-485 nm emmission, and the F-actin stained with Alexa Fluor 488 was visualised under 488 nm excitation and 510-560 nm emmission. Nanolive (Nanolive SA, Lausanne, Switzerland) was used to measure refractive indices and to reconstruct 3-dimensional refractive index images of the cells after treatment with PtNPs.

*Analysis of Formation of Protein Coronas*

Fresh blood sample was withdrawn aseptically by antecubital venipuncture of healthy human donor with signed informed consent. Immediately after collection, plasma was isolated from whole blood by centrifugation (3000×g, 5 min). Subsequently, 1 mg/mL of PtNPs were incubated in plasma at 1:1 ratio (v/v). The incubation was done for 45 min at 37 °C under 300×g. The protein corona-bound nanoparticles were recovered after 10 min centrifugation at 15 000×g and washed three times in cold PBS to remove unbound proteins. Finally, the proteins were eluted by adding sodium dodecyl sulfate (SDS), separated by 12,5 % 1D SDS-polyacrylamide gel electrophoresis (PAGE) and stained by Coomassie Brilliant Blue. Gels were visualized using Azure c600 (Azure Biosystems, Dublin, CA, USA).

*Single cell gel electrophoresis (Comet assay)*

A six-well plate was plated with cells at a density of 10^6^ cells/well and treated with PtNPs (IC_50_ concentration calculated from MTT assay) for 24 h. H_2_O_2_ (150 μM) was use as a control. After harvesting, approximately 15 μL of the cell suspension was mixed with 75 mL of 0.5% low melting point agarose (CLP, San Diego, CA, USA) and layered on one end of a frosted plain glass slide. Then it was covered with a layer of the low melting agarose. After solidification of the gel, the slides were immersed in lysing solution (2.5 M NaCl, 100 mM Na_2_EDTA, 10 mM Tris, pH 10) containing 1% Triton X-100 and 10% DMSO for overnight incubation at 4 °C. A cold alkaline electrophoresis buffer was poured into the chamber and incubated for 30 min at 4 °C. The electrophoresis was carried at 4 °C for 30 min, at 1.25 V/cm. After the neutralization of the slides with 0.4 M Tris, pH 7.5 they were stained with ethidium bromide (EtBr, 2 mg/mL). The cells were analysed using a fluorescence microscope EVOS FL Auto Cell Imaging System (Thermo Fisher Scientific, Waltham, MA, USA) and Open-Comet software and Comet Score version 1.3 plug-in in ImageJ software version 1.52a was used to analyze the obtained images. The level of DNA damage was expressed through the tail moment presenting the percentage of DNA in the tail (Gyori et al., 2014).

*Total ROS determination*

For analysis of total ROS, HaCaT or HEK293 cell (~1×10^6^ per well) were seeded into flat bottom tissue culture plates and incubated overnight to allow attachment. Subsequently, cells were exposed to annotated concentration of 50 µg/mL of PtNPs-10, PtNPs-40 or CDDP and incubated for another 24 h. Then, the cells were washed twice with phosphate-buffered saline (PBS, pH 7.4) and incubated with 10 µM 2',7'-dichlorodihydrofluorescein diacetate (H_2_DCFDA; Invitrogen, USA) for 30 min (the incubation steps were conducted in a 5% CO_2_-humidified atmosphere at 37 ◦C). After the incubation period cells were harvested by centrifugation (300×*g*, 5 min, 4°C) and fluorescence intensity analyzed using the BD Accuri C6 Plus (BD Biosciences, USA). Dead cells were gated out by 7-aminoactinomycin D (7-AAD; Exbio, Czech Republic) staining. For each experiment, at least 25,000 events/sample were recorded. Data were analyzed using FlowJo software (FlowJo; RRID: SCR_008520). The analysis was done in duplicate, and two independent experiments were performed.

*LC–MS/MS quantification of selected metabolites of TCA cycle and methionine cycle*

The determination of the individual metabolites involved in TCA cycle and methionine cycle were determined using liquid chromatography and tandem mass spectrometry with triple-quadrupole MS detector according to Kolcakova et al. (Kolackova et al., 2020). Agilent 1200 Rapid Resolution LC system was connected to an Agilent Technologies 6460 triple-quadrupole MS detector with an Agilent Jet Stream all from Agilent Technologies, Waldbronn, Germany. The metabolites were extracted with 1mL of 0.1M HCl, and for the separation, a Zorbax EC18 (Technologies, Palo Alto, CA, USA) and Agilent chromatography column (4.6 × 100 mm, 2.7 μm) were used.

*Chorioallantoic Membrane Assay*

The incubator RCom 50 MAX (Korea) with controlled environmental conditions (temperature of 37.5°C and humidity control of 55% rH) was used for incubation of Lankenfeld roosters and ISA Brown hens fertilized eggs (Integra, a.s., Czech Republic). The vitality of chicken embryos was monitored by digital egg monitoring system Avitronics (Vetronic services, England). At the 7^th^ day of development, the solutions of PtNPs-10, PtNPs-40 and CDDP (100 µL; 37.5 – 300 µg/mL) were applied through small hole in egg shell into the air cell on the chorioallantoic membrane using Chirana T. injection (maximal volume: 1 mL, size: 0.33 x 12 mm). The hole was covered by paraffin. After 10 days of the incubation with the given treatment, the liver and kidney were taken for analysis. In the European Union countries, CAM assay is not considered an animal experiment and, therefore, it does not require ethical approval.

*Acidic hydrolysis of liver or kidney samples*

The 10 mg of liver or kidney samples was mixed with 0.5 mL of 6 M HCl and solution was subsequently subjected to digestion in a microwave reactor Anton Paar (Anton Paar GmbH, Graz, Austria) under controlling conditions for 90 min (power 80 W, temperature 120 °C and maximum pressure of 25 bar). After acidic hydrolysis samples were centrifuged (Centrifuge Z326 K, Hermile, Germany) at 4 °C, 24 000 g, 10 min Further, the 100 µL of hydrolyzed sample of liver or kidney was diluted with 900 µL of dilution buffer composed of 5 mL·L^−1^ of thiodiglycol, 14 g·L^−1^ of citric acid, 11.5 g·mL^−1^ of sodium chloride and centrifuged (Centrifuge Z326 K, Hermile, Germany) at 4 °C, 24 000 g, 10 min. After this procedure sample was diluted with 500µL of 0.6M NaOH in the dilution buffer and subsequently used for analysis by ion-exchange chromatography.

Ion-Exchange Liquid Chromatography (IELC) Analyses of Amino Acids

The determination of amino acids (AA) content in cells and tissues before and after application of CDDP, PtNPs-10 and PtNPs-40 was conducted by IELC. The IELC (Model AAA-400, Ingos, Prague, Czech Republic) equiped with UV/VIS light absorption detector and post column derivatization by ninhydrin were used for analysis of amino acids profile in samples. The glass column with inner diameter of 3.7 mm and 350 mm length was filed manually with strong cation exchanger in the sodium cycle LG ANB. The average size of particles was around 12 μm with 8% of porosity. The double channel UV/VIS light absorption detector was set to two wavelengths of 440 and 570 nm and the working temeperature of column was set on 60 °C. Solution of ninhydrin was prepared in 75% v/v methylcelosolve (Ingos, Prague, Czech Republic) and in 2% v/v 4 M acetic buffer (pH 5.5). Tin chloride (SnCl_2_) was used as a reducing agent. Elution of amino acid was done by a buffer containing 10.0 g of citric acid, 5.6 g of sodium citrate, and 8.36 g of NaCl per liter of solution and pH was 3.0. Flow rate was 0.25 mL min^−1^. Reactor temperature was set on 120 °C. For dilution of samples, a dilution buffer was used (composition: thiodiglycol 5 mL L ^−1^ , citric acid 14 g L ^−1^ , sodium chloride 11.5 g mL^−1^).

Statistical analysis

Descriptive statistics and general regression model statistics was used to analyze data, where “organ” and “platinum drug” were used as independent categorical predictors and “concentration” was used as independent continuous predictor. One-way ANOVA and multivariate ANOVA with covariance (MANCOVA) was applied for the analysis of the *in vitro* and *in vivo* data respectively, with Tuckey HSD post-hoc analysis included. A “p” level ≤ 0.05 was considered significant unless indicated otherwise. Next, principal component analysis (PCA) was performed on non-standardized data to determine the relationship between the selected amino acids and treatments. Categorical principal component analysis was applied on *in vitro* data, whereas PCA was applied for *in vitro* data for each treatment separately. All statistical analyses were performed using IBM SPSS Statistics Software, Version 26.

**Results**


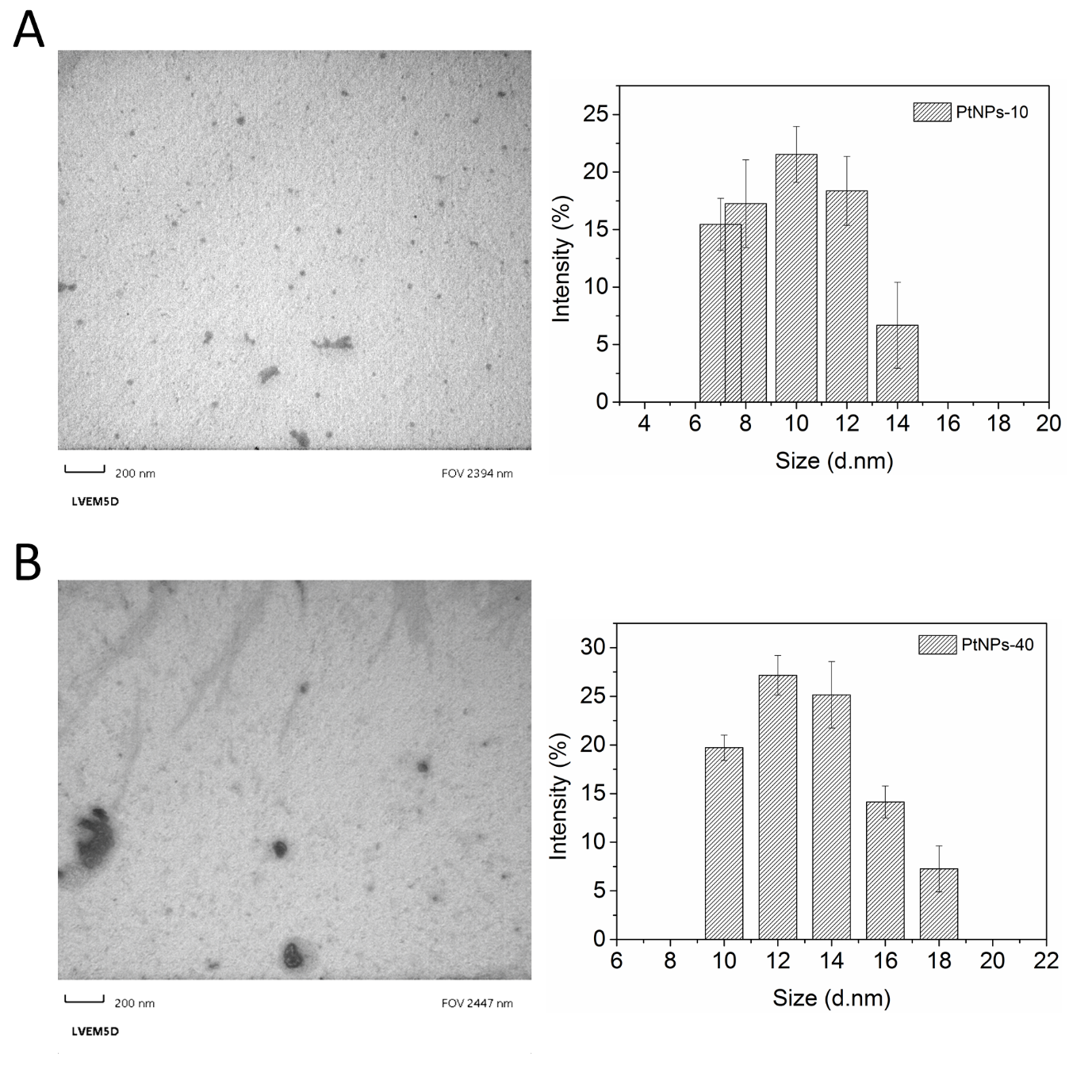


Figure S1. Characterization of PtNPs-10 and PtNPs-40 using TEM with a magnification of 200 kx; scale bar, 200 nm. The size and surface charge were obtained by DLS measurement using a detector angle of 173° and wavelength of 633 nm. A) TEM images showing the uniformity and good dispersity of successfully synthetized PtNPs-10 and a graph representing the size


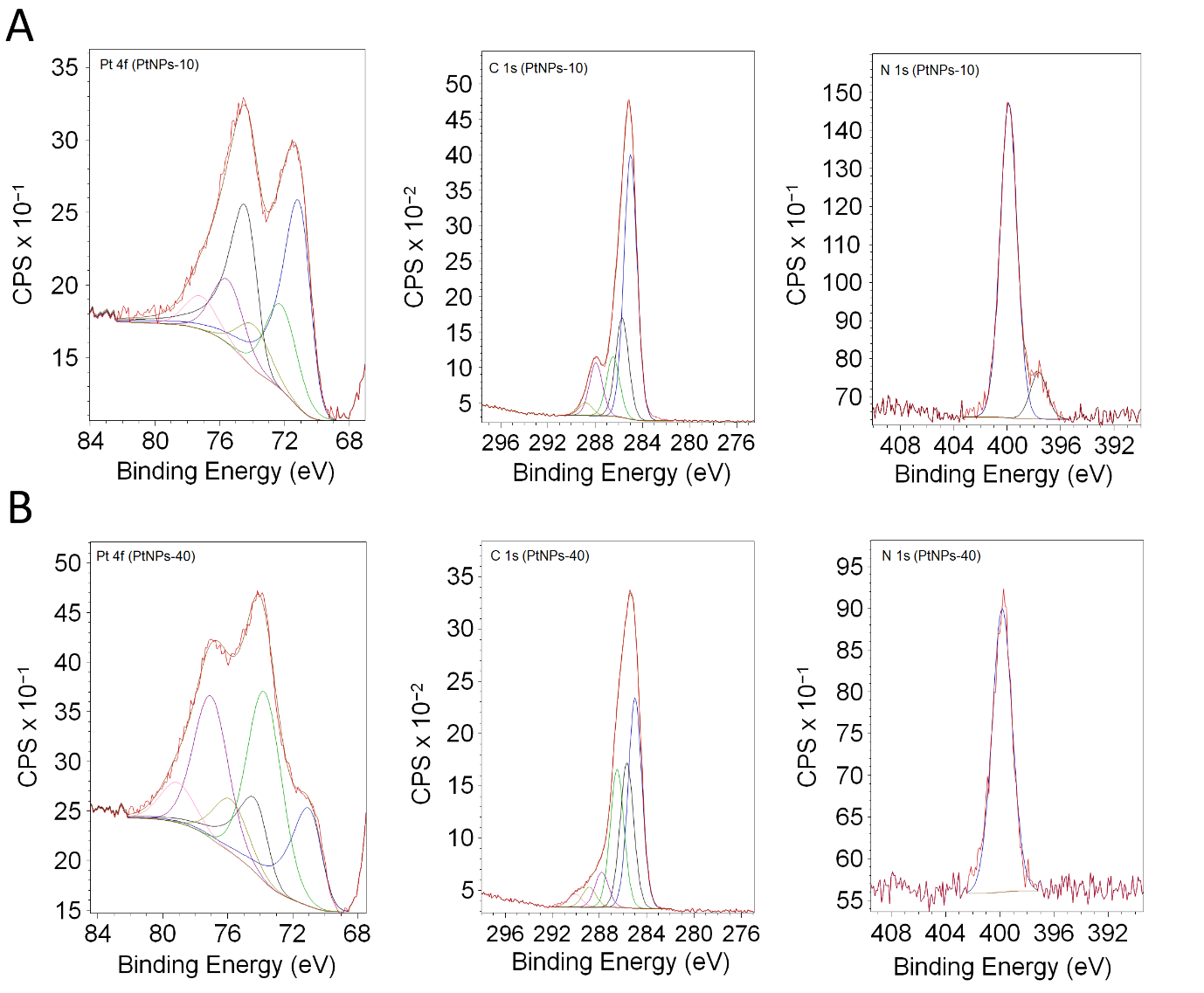


Figure S2. X-ray photoelectron spectroscopy spectra measured by spectrometer operated at 150 W using a monochromatic Al Kα (hν = 1486.7 eV) X-ray source with area analysis of ~300 × 700 µm. A) High-resolution Pt 4f, C 1s and N 1s spectra obtained during chemical composition analysis of PtNPs-10; B) High-resolution Pt 4f, C 1s and N 1s spectra obtained during chemical composition analysis of PtNPs-40.


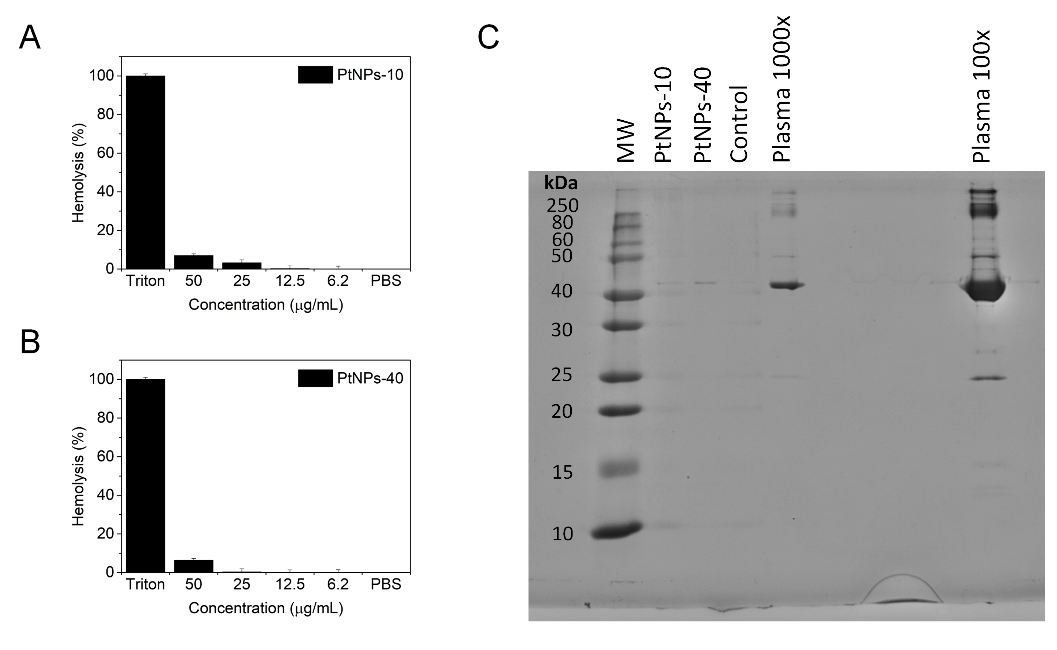


Figure S3. Hemocompatibility assessment of PtNPs and interaction with plasma proteins. A) Hemocompatibility of PtNPs-10 assayed on human RBCs. Positive and negative controls were measured using 0.1% Triton X-100 and PBS (pH 7.4), respectively; B) Hemocompatibility of PtNPs-40 assayed on human RBCs. Positive and negative controls were measured using 0.1% Triton X-100 and PBS (pH 7.4), respectively; C) SDS-PAGE showing the profiles of protein corona formation obtained after incubation of human plasma with PtNPs.


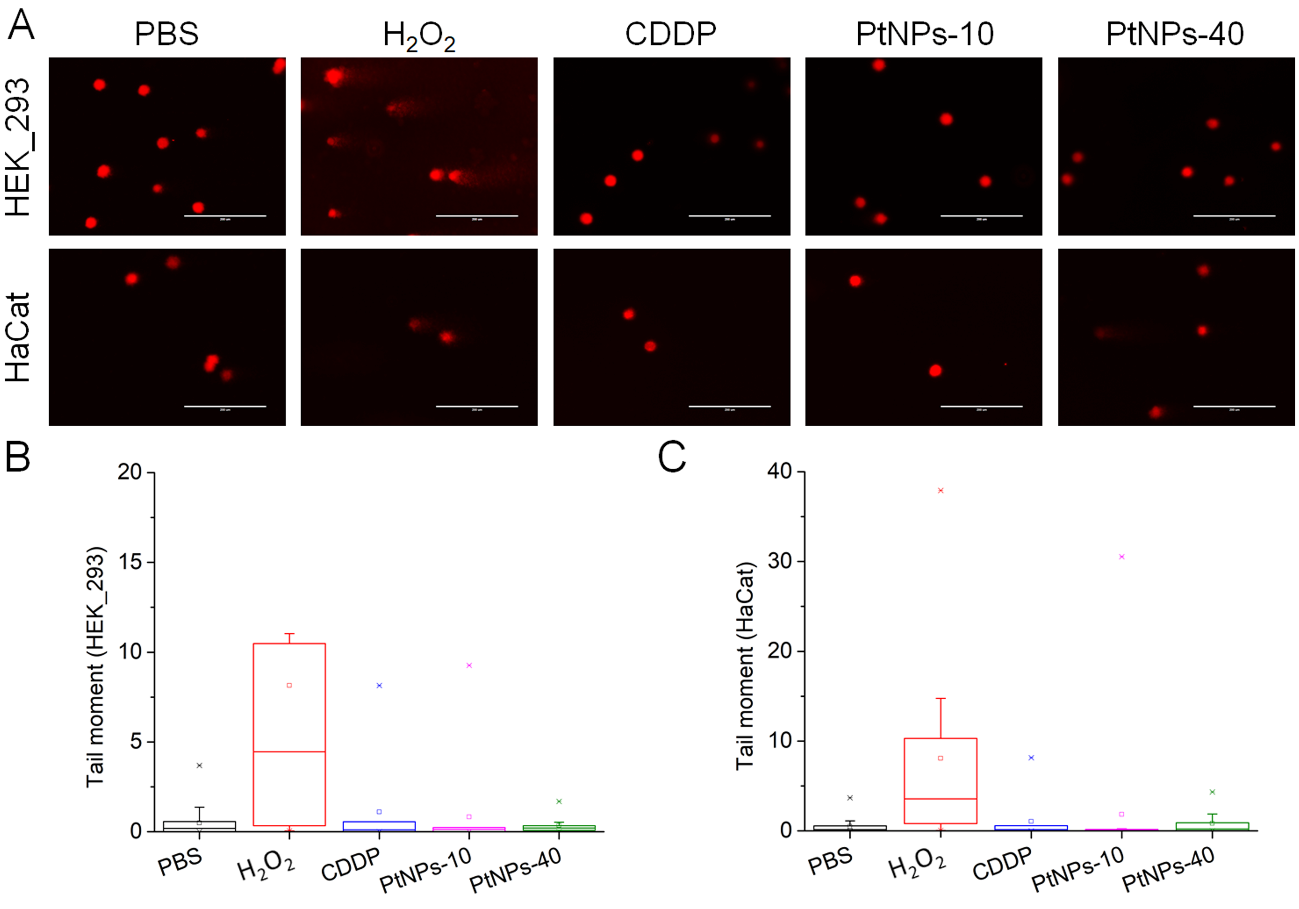


Figure S4. A) Comet assay tested on HEK-293 and HaCat cell lines showing the DNA stability or strands breakage and loss of supercoiled structure after treatment with PtNPs and CDDP. PBS buffer with pH 7.4 was used as negative control while H_2_O_2_ was a positive control; B) Quantitation of the level of DNA damage in HEK-293 cell line, evaluated after the exposure to PtNPs and CDDP and presented as percentage of DNA in the tail of the comet (tail moment); C) Quantitation of the level of DNA damage in HaCat cell line, evaluated after the exposure to PtNPs and CDDP and present as percentage of DNA in the tail of the comet (tail moment).

Table S1. Level of significance (p value) of the differences between the amino acids of the cell lines (HaCat and HEK-293) and between tissues (kidney and liver) in each group (control, CDDP, PtNPs-10 and PtNPs-40). Significant differences are shown at p ≤ 0.05

|  | HaCat vs. HEK-293 | | | | Liver vs. Kidney | | | |
| --- | --- | --- | --- | --- | --- | --- | --- | --- |
| AA | Control | CDDP | PtNPs-10 | PtNPs-40 | Control | CDDP | PtNPs-10 | PtNPs-40 |
| Asp | **0,000*** | **0,000*** | **0,001*** | **0,000*** | 0,369 | 0,111 | **0,005*** | 0,009 |
| Thr | **0,000*** | **0,000*** | **0,000*** | **0,000*** | 0,617 | 0,499 | **0,009*** | 0,063 |
| Ser | **0,001*** | **0,000*** | **0,001*** | **0,000*** | 0,895 | 0,462 | 0,051 | 0,072 |
| Glu | **0,000*** | **0,000*** | **0,000*** | **0,000*** | 0,596 | 0,262 | 0,178 | 0,014 |
| Pro | **0,010*** | 0,164 | **0,037*** | **0,032*** | 0,332 | 0,621 | 0,157 | 0,217 |
| Gly | **0,000*** | **0,000*** | **0,000*** | **0,000*** | 0,883 | 0,240 | 0,910 | **0,002*** |
| Ala | **0,000*** | **0,000*** | **0,000*** | **0,000*** | 0,725 | 0,099 | 0,103 | 0,090 |
| Cys | **0,000*** | **0,000*** | **0,000*** | **0,000*** | 0,167 | 0,081 | **0,035*** | 0,628 |
| Val | 0,153 | 0,102 | **0,001*** | 0,233 | 0,090 | **0,006*** | 0,836 | 0,119 |
| Met | **0,000*** | **0,000*** | **0,000*** | **0,000*** | 0,695 | 0,822 | 0,082 | 0,860 |
| Ile | **0,000*** | **0,000*** | **0,000*** | **0,000*** | 0,130 | **0,027*** | **0,025*** | 0,367 |
| Leu | 0,358 | **0,001*** | 0,121 | **0,000*** | 0,094 | **0,002*** | 0,548 | **0,002*** |
| Tyr | **0,000*** | **0,000*** | **0,017*** | **0,000*** | 0,995 | 0,916 | 0,511 | 0,890 |
| Phe | 0,065 | **0,000*** | **0,001*** | **0,000*** | 0,640 | 0,736 | **0,014*** | 0,141 |
| His | **0,000*** | **0,000*** | **0,000*** | **0,000*** | 0,101 | 0,087 | **0,008*** | 0,057 |
| Lys | 0,383 | **0,019*** | 0,463 | 0,052 | 0,491 | 0,292 | 0,097 | **0,009*** |
| Arg | 0,689 | **0,000*** | **0,000*** | **0,000*** | 0,183 | **0,006*** | 0,237 | **0,004*** |

Table S2. Weights of chicken embryo, liver and kidneys expressed as mean values (g) ± SD. Significant differences between the weights of the embryos and between the weights of each tissue (kidney and liver) upon the treatments (CDDP, PtNPs‑10 and PtNPs-40) compared to the untreated embryos are shown at p ≤ 0.05.

|  | Mean ± SD  (g)  Control | Mean ± SD (g)  CDDP | Mean ± SD  (g) PtNPs-10 | Mean ± SD (g)  PtNPs-40 | Control  vs.  CDDP | Control  vs.  PtNPs-10 | Control  vs.  PtNPs-40 | CDDP  vs.  PtNPs-10 | CDDP  vs.  PtNPs-40 | PtNPs-10  vs.  PtNPs-40 |
| --- | --- | --- | --- | --- | --- | --- | --- | --- | --- | --- |
| Embryo weight | 19.16 ± 0.51 | 16.90 ± 0.75 | 17.05 ± 0.39 | 17.16 ± 0.49 | **0,000*** | **0,000*** | **0,000*** | 0,979 | 0,713 | 0,914 |
| Kidney weight | 0.09 ± 0.00 | 0.07 ± 0.01 | 0.07 ± 0.01 | 0.07 ± 0.01 | **0,000*** | **0,000*** | **0,000*** | 0,999 | 0,73 | 0,666 |
| Liver weight | 1.76 ± 0.08 | 1.27 ± 0.06 | 1.48 ± 0.06 | 1.38 ± 0.10 | **0,000*** | **0,000*** | **0,000*** | **0,000*** | **0,009*** | **0,044*** |

Reference

Buchtelova, H., Dostalova, S., Michalek, P., Krizkova, S., Strmiska, V., Kopel, P., et al. (2017). Size-related cytotoxicological aspects of polyvinylpyrrolidone-capped platinum nanoparticles. *Food and Chemical Toxicology* 105**,** 337-346. doi: 10.1016/j.fct.2017.04.043.

Evans, B.C., Nelson, C.E., Yu, S.S., Beavers, K.R., Kim, A.J., Li, H., et al. (2013). Ex vivo red blood cell hemolysis assay for the evaluation of pH-responsive endosomolytic agents for cytosolic delivery of biomacromolecular drugs. *J Vis Exp* (73)**,** e50166. doi: 10.3791/50166.

Gyori, B.M., Venkatachalam, G., Thiagarajan, P.S., Hsu, D., and Clement, M.V. (2014). OpenComet: an automated tool for comet assay image analysis. *Redox Biol* 2**,** 457-465. doi: 10.1016/j.redox.2013.12.020.

Heger, Z., Gumulec, J., Cernei, N., Tmejova, K., Kopel, P., Balvan, J., et al. (2015). 17beta-estradiol-containing liposomes as a novel delivery system for the antisense therapy of ER-positive breast cancer: An in vitro study on the MCF-7 cell line. *Oncol Rep* 33(2)**,** 921-929. doi: 10.3892/or.2014.3627.

Kolackova, M., Chaloupsky, P., Cernei, N., Klejdus, B., Huska, D., and Adam, V. (2020). Lycorine and UV-C stimulate phenolic secondary metabolites production and miRNA expression in Chlamydomonas reinhardtii. *J Hazard Mater* 391**,** 122088. doi: 10.1016/j.jhazmat.2020.122088.
